# *TMEM175*, *SCARB2* and *CTSB* associations with Parkinson’s disease risk across populations

**DOI:** 10.1038/s41531-025-01180-z

**Published:** 2025-12-02

**Authors:** Wenhua Sun, Claudia Schulte, Thomas Gasser, Manuela Tan, the Global Parkinson’s Genetic Program (GP2), Lasse Pihlstrøm, Pedro Chana, Yeajin Song, Sara Bandres-Ciga, Cornelis Blauwendraat, Andrew Singleton, Mike A. Nalls, Hampton Leonard, Mie Rizig, Hirotaka Iwaki, Carlos Rieder, Ignacio F. Mata, Njideka Okubadejo, Emilia M. Gatto, Marcelo Kauffman, Claire E. Shepherd, Samson Khachatryan, Zaruhi Tavadyan, Julie Hunter, Kishore Kumar, Melina Ellis, Miguel E. Rentería, Sulev Koks, Alexander Zimprich, Vitor Tumas, Sarah Camargos, Edward A. Fon, Ted Fon, Oury Monchi, Benjamin Pizarro Galleguillos, Patricio Olguin, Marcelo Miranda, Maria Leonor Bustamante, Beisha Tang, Huifang Shang, Jifeng Guo, Piu Chan, Wei Luo, Gonzalo Arboleda, Jorge Orozco, Marlene Jimenez del Rio, Alvaro Hernandez, Mohamed Salama, Walaa A. Kamel, Yared Z. Zewde, Alexis Brice, Jean-Christophe Corvol, Ana Westenberger, Christine Klein, Eva-Juliane Vollstedt, Harutyun Madoev, Joanne Trinh, Johanna Junker, Katja Lohmann, Anastasia Illarionova, Brit Mollenhauer, Franziska Hopfner, Günter Höglinger, Lara M. Lange, Manu Sharma, Sergio Groppa, Zih-Hua Fang, Albert Akpalu, Georgia Xiromerisiou, Georgios Hadjigeorgiou, Efthymios Dardiotis, Ioannis Dagklis, Ioannis Tarnanas, Leonidas Stefanis, Maria Stamelou, Alex Medina, Germaine Hiu-Fai Chan, Nelson Yuk-Fai Cheung, Nancy Ip, Phillip Chan, Xiaopu Zhou, Asha Kishore, K. P. Divya, Pramod Pal, Prashanth Lingappa Kukkle, Roopa Rajan, Rupam Borgohain, Mehri Salari, Andrea Quattrone, Enza Maria Valente, Micol Avenali, Lucilla Parnetti, Tommaso Schirinzi, Manabu Funayama, Nobutaka Hattori, Tomotaka Shiraishi, Altynay Karimova, Gulnaz Kaishibayeva, Cholpon Shambetova, Rejko Krüger, Ai Huey Tan, Azlina Ahmad-Annuar, Shen-Yang Lim, Yi Wen Tay, Mohamed Ibrahim Norlinah, Shahrul Azmin, Nor Azian Abdul Murad, Daniel Martinez-Ramirez, Mayela Rodriguez-Violante, Paula Reyes-Pérez, Bayasgalan Tserensodnom, Rajeev Ojha, Tim J. Anderson, Toni L. Pitcher, Oluwadamilola Ojo, Jan O. Aasly, Shoaib Ur-Rehman, Mario Cornejo-Olivas, Maria Leila Doquenia, Raymond Rosales, Angel Vinuela, Elena Iakovenko, Bashayer Al Mubarak, Muhammad Umair, Eng-King Tan, Ferzana Amod, Jonathan Carr, Soraya Bardien, Beomseok Jeon, Yun Joong Kim, Esther Cubo, Ignacio Alvarez, Janet Hoenicka, Katrin Beyer, Pau Pastor, Sarah El-Sadig, Christiane Zweier, Paul Krack, Chin-Hsien Lin, Ruey-Meei Wu, Hsiu-Chuan Wu, Yih-Ru Wu, Pin-Jui Kung, Serena Wu, Rim Amouri, Samia Ben Sassi, A. Nazl Başak, Özgür Öztop Çakmak, Sibel Ertan, Gencer Genc, Alejandro Martínez-Carrasco, Anette Schrag, Anthony Schapira, Eleanor J. Stafford, Henry Houlden, Huw R. Morris, John Hardy, Nicholas Wood, Olaitan Okunoye, Rauan Kaiyrzhanov, Rimona Weil, Simona Jasaitye, Vida Obese, Camille Carroll, Claire Bale, Donald Grosset, Kin Y. Mok, Nigel Williams, Patrick A. Lewis, Seth Love, Simon Stott, Alberto Espay, Luca Marsili, Alyssa O’Grady, Bernadette Siddiqi, Bradford Casey, Brian Fiske, Charisse Comart, Justin C. Solle, Kaileigh Murphy, Maggie Kuhl, Naomi Louie, Sohini Chowdhury, Todd Sherer, Andrew K. Sobering, Cabell Jonas, Carlos Cruchaga, Caroline B. Pantazis, Claire Wegel, Deborah Hall, Ejaz Shamim, Jared Williamson, Ekemini Riley, Sonya Dumanis, Geidy E. Serrano, Thomas Beach, Honglei Chen, Ignacio Juan Keller Sarmiento, Niccolò E. Mencacci, Steven Lubbe, Joseph Jankovic, Miguel Inca-Martinez, Joshua Shulman, Karen Nuytemans, Karl Kieburtz, Katerina Markopoulou, Kenneth Marek, Lana M. Chahine, Lauren Ruffrage, Marissa Dean, Lisa Shulman, Roger Albin, Roy Alcalay, Ruth Walker, Tao Xie, Tatiana Foroud, Duan Nguyen, Toan Nguyen, Masharip Atadzhanov

**Affiliations:** 1 Department of Neurodegenerative Diseases, Hertie Institute for Clinical Brain Research, University of Tuebingen and German Center for Neurodegenerative Diseases (DZNE), Tuebingen, Germany; 2 Department of Neurology, Oslo University Hospital, Oslo, Norway; 3 Centro de Trastornos del Movimiento (CETRAM), Santiago, Chile; 4 Centre for Alzheimer’s and Related Dementias. National Institute on Aging, National Institutes of Health, Bethesda, MD USA; 5 Laboratory of Neurogenetics, National Institute on Aging, National Institutes of Health, Bethesda, MD USA; 6 DataTecnica LLC, Washington, DC USA; 7 Department of Neuromuscular Diseases, UCL Queen Square Institute of Neurology, London, UK; 8 Preventive Neurology Unit, Wolfson Institute of Population Health, Queen Mary University of London, London, UK; 9 Department of Anatomy, College of Medicine, University of Lagos, Lagos, Nigeria; 10 Serviço de Neurologia, Hospital de Clínicas de Porto Alegre, Porto Alegre, Brazil; 11 Universidade Federal do Rio Grande do Sul / Hospital de Clínicas de Porto Alegre, Porto Alegre, Brazil; 12 Genomic Medicine, Lerner Research Institute, Cleveland Clinic Foundation, Cleveland, OH USA; 13 Department of Medicine, College of Medicine, University of Lagos, Lagos, Nigeria; 14 Sanatorio de la Trinidad Mitre-INEBA, Buenos Aires, Argentina; 15 Hospital JM Ramos Mejia, Buenos Aires, Argentina; 16 Neuroscience Research Australia, Sydney, NSW Australia; 17 Somnus Neurology Clinic, Yerevan, Armenia; 18 ANZAC Research Institute, Concord, NSW Australia; 19 Garvan Institute of Medical Research and Concord Repatriation General Hospital, Darlinghurst, NSW Australia; 20 Concord Hospital, Concord, NSW Australia; 21 QIMR Berghofer Medical Research Institute, Herston, QLD Australia; 22 Murdoch University, Perth, WA Australia; 23 Medical University of Vienna, Vienna, Austria; 24 University of São Paulo, São Paulo, Brazil; 25 Universidade Federal de Minas Gerais, Belo Horizonte, Brazil; 26 McGill University, Montreal, QC Canada; 27 Universidad de Chile, Santiago, Chile; 28 Fundación Diagnosis, Santiago, Chile; 29 Faculty of Medicine, Universidad de Chile, Santiago, Chile; 30 CETRAM, Santiago, Chile; 31 Central South University, Changsha, China; 32 West China Hospital, Sichuan University, Chengdu, China; 33 Xiangya Hospital, Central South University, Changsha, China; 34 Capital Medical University, Beijing, China; 35 Zhejiang University, Hangzhou, China; 36 Universidad Nacional de Colombia, Bogotá, Colombia; 37 Fundación Valle del Lili, Santiago de Cali, Colombia; 38 University of Antioquia, Medellín, Colombia; 39 University of Costa Rica, San José, Costa Rica; 40 The American University in Cairo, Cairo, Egypt; 41 Beni-Suef University, Beni Suef, Egypt; 42 Addis Ababa University, Addis Ababa, Ethiopia; 43 Paris Brain Institute, Paris, France; 44 Sorbonne Université, Paris, France; 45 University of Lübeck, Lübeck, Germany; 46 Deutsches Zentrum für Neurodegenerative Erkrankungen (DZNE), Göttingen, Germany; 47 University Medical Center Göttingen, Göttingen, Germany; 48 University Hospital LMU Munich, Munich, Germany; 49 Hannover Medical School, Hannover, Germany; 50 University of Lübeck and University Medical Center Schleswig-Holstein, Lübeck, Germany; 51 University of Tübingen, Tübingen, Germany; 52 The German Center for Neurodegenerative Diseases (DZNE), Göttingen, Germany; 53 University of Ghana Medical School, Accra, Ghana; 54 University of Thessaly, Larissa, Greece; 55 Aristotle University of Thessaloniki, Thessaloniki, Greece; 56 Ionian University, Corfu, Greece; 57 Biomedical Research Foundation of the Academy of Athens, Athens, Greece; 58 Diagnostic and Therapeutic Centre HYGEIA Hospital, Marousi, Greece; 59 Hospital San Felipe, Tegucigalpa, Honduras; 60 Queen Elizabeth Hospital, Kowloon, Hong Kong; 61 The Hong Kong University of Science and Technology, Kowloon, Hong Kong; 62 Aster Medcity, Kochi, India; 63 Sree Chitra Tirunal Institute for Medical Sciences and Technology, Thiruvananthapuram, India; 64 National Institute of Mental Health & Neurosciences (NIMHANS), Bengaluru, India; 65 Manipal Hospital, Delhi, India; 66 All India Institute of Medical Sciences, Delhi, India; 67 Nizam’s Institute of Medical Sciences, Hyderabad, India; 68 Shahid Beheshti University of Medical Sciences, Tehran, Iran; 69 Magna Græcia University of Catanzaro, Catanzaro, Italy; 70 University of Pavia, Pavia, Italy; 71 University of Perugia, Perugia, Italy; 72 University of Rome Tor Vergata, Rome, Italy; 73 Juntendo University, Tokyo, Japan; 74 Juntendo University Faculty of Medicine, Tokyo, Japan; 75 Jikei University School of Medicine, Tokyo, Japan; 76 Institute of Neurology and Neurorehabilitation, Almaty, Kazakhstan; 77 Kyrgyz State Medical Academy, Bishkek, Kyrgyzstan; 78 Luxembourg Centre for Systems Biomedicine, University of Luxembourg, Belvaux, Luxembourg; 79 University of Malaya, Kuala Lumpur, Malaysia; 80 Universiti Kebangsaan Malaysia, Kuala Lumpur, Malaysia; 81 UKM Medical Molecular Biology Institute (UMBI), Kuala Lumpur, Malaysia; 82 Universiti Kebangsaan Malaysia Medical Centre, Kuala Lumpur, Malaysia; 83 Tecnológico de Monterrey, Monterrey, Mexico; 84 Instituto Nacional de Neurología y Neurocirugía, Mexico City, Mexico; 85 Universidad Nacional Autónoma de México, Mexico City, Mexico; 86 Mongolian National University of Medical Sciences, Ulaanbaatar, Mongolia; 87 Tribhuvan University, Kirtipur, Nepal; 88 University of Otago, Christchurch, New Zealand; 89 University of Otago, Dunedin, New Zealand; 90 College of Medicine, University of Lagos, Lagos, Nigeria; 91 Norwegian University of Science and Technology, Trondheim, Norway; 92 University of Science and Technology Bannu, Khyber Pakhtunkhwa, Pakistan; 93 Instituto Nacional de Ciencias Neurológicas, Lima, Peru; 94 Metropolitan Medical Center, Manila, Philippines; 95 University of Puerto Rico, San Juan, Puerto Rico; 96 Research Center of Neurology, Moscow, Russia; 97 King Faisal Specialist Hospital and Research Center, Riyadh, Saudi Arabia; 98 King Abdullah International Medical Research Center, Jeddah, Saudi Arabia; 99 National Neuroscience Institute, Singapore, Singapore; 100 University of KwaZulu-Natal, Durban, South Africa; 101 University of Stellenbosch, Stellenbosch, South Africa; 102 Stellenbosch University, Stellenbosch, South Africa; 103 Seoul National University Hospital, Seoul, South Korea; 104 Yongin Severance Hospital, Seoul, South Korea; 105 Hospital Universitario de Burgos, Burgos, Spain; 106 University Hospital Mutua Terrassa, Barcelona, Spain; 107 Institut de Recerca Sant Joan de Déu, Barcelona, Spain; 108 Research Institute Germans Trias i Pujol, Badalona, Spain; 109 University Hospital Germans Trias i Pujol, Badalona, Spain; 110 Faculty of Medicine, University of Khartoum, Khartoum, Sudan; 111 Inselspital, Bern University Hospital, University of Bern, Bern, Switzerland; 112 National Taiwan University Hospital, Taipei City, Taiwan; 113 Chang Gung Memorial Hospital, Taoyuan City, Taiwan; 114 National Taiwan University, Taipei City, Taiwan; 115 Chang Gung University, Taoyuan City, Taiwan; 116 National Institute Mongi Ben Hamida of Neurology, Tunis, Tunisia; 117 Mongi Ben Hmida National Institute of Neurology, Tunis, Tunisia; 118 Koç University, Istanbul, Turkey; 119 Şişli Etfal Training and Research Hospital, Istanbul, Turkey; 120 University College London, London, UK; 121 University of Plymouth, Plymouth, UK; 122 Parkinson’s UK, London, UK; 123 University of Glasgow, Glasgow, UK; 124 Queen Mary University of London, London, UK; 125 Cardiff University, Cardiff, UK; 126 Royal Veterinary College, University of London, London, UK; 127 University of Bristol, Bristol, UK; 128 Cure Parkinson’s Trust, London, UK; 129 University of Cincinnati, Cincinnati, OH USA; 130 The Michael J. Fox Foundation for Parkinson’s Research, New York, NY USA; 131 Augusta University / University of Georgia Medical Partnership, Athens, GA USA; 132 Mid-Atlantic Permanente Medical Group, Rockville, MD USA; 133 Washington University in St. Louis, St. Louis, MO USA; 134 National Institutes of Health, Bethesda, MD USA; 135 Indiana University, Bloomington, IN USA; 136 Rush University Medical Center, Chicago, IL USA; 137 Kaiser Permanente, Oakland, CA USA; 138 Aligning Science Across Parkinson’s, Washington, DC USA; 139 Banner Sun Health Research Institute, Sun City, AZ USA; 140 Michigan State University, East Lansing, MI USA; 141 Northwestern University, Chicago, IL USA; 142 Baylor College of Medicine, Houston, TX USA; 143 Baylor College of Medicine/Texas Children’s Hospital, Houston, TX USA; 144 University of Miami Miller School of Medicine, Miami, FL USA; 145 Beth Israel Deaconess Medical Center, Boston, MA USA; 146 NorthShore University HealthSystem, Evanston, IL USA; 147 Institute for Neurodegenerative Disorders, New Haven, CT USA; 148 University of Pittsburgh, Pittsburgh, PA USA; 149 University of Alabama at Birmingham, Birmingham, AL USA; 150 University of Maryland School of Medicine, Baltimore, MD USA; 151 University of Michigan, Ann Arbor, MI USA; 152 Columbia University Irving Medical Center, New York, NY USA; 153 James J. Peters VA Medical Center, Bronx, NY USA; 154 University of Chicago, Chicago, IL USA; 155 Indiana University School of Medicine, Indianapolis, IN USA; 156 Hue University, Huế, Vietnam; 157 University of Zambia, Lusaka, Zambia

**Keywords:** Diseases, Genetics, Neurology, Neuroscience

## Abstract

Genome-wide association study of Parkinson’s disease (PD) identified common variants associated with lysosomal mechanism, including *TMEM175, SCARB2*, and *CTSB*. We investigated the association between common and rare variants across populations using cohorts from the Global Parkinson’s Genetics Program (GP2) (33,733 cases and 18,703 controls from ten ancestries). In the European cohort, we confirmed significant associations with PD risk for all known genetic risk variants across the three genes and *TMEM175* p. Met393Thr as an independent genome-wide significant signal. Additionally, a novel independent signal, *SCARB2* rs11547135, was detected. The burden analysis linked PD to *SCARB2* in African American, Ashkenazi Jewish and East Asian cohorts. Single variants-based tests identified rare missense variants in *SCARB2* in several populations. Our study reinforces the association of lysosomal genetic variants with PD risk, revealing genetic heterogeneity across populations.

## Introduction

Glucocerebrosidase (GCase), encoded by *GBA1*, is a lysosomal enzyme critical for maintaining lysosomal protein and lipid homeostasis. GCase deficiency causes accumulation of alpha-synuclein(α-syn)^1^ and variants in *GBA1* are recognized as a common genetic risk factor for PD^2^. The latest genome-wide association study (GWAS) in European derived populations identified common genetic variants associated with lysosomal dysfunction that contribute to Parkinson’s disease (PD) risk, with *GBA1* (2025 PD GWAS, p.E365K (rs2230288, odds ratio [OR] = 1.66)) emerging as a key locus^3–5^. Genetic variants in other lysosome-related genes (including transmembrane protein 175 [*TMEM175*], Scavenger Receptor Class B Member 2 [*SCARB2*] and Cathepsin B [*CTSB*]) are also associated with PD risk and age at onset^6–8^. These candidate genes were prioritized in this study based on (1) *GBA1*’s role as a major lysosomal driver of PD pathogenesis; (2) functional convergence of *TMEM175*, *SCARB2*, and *CTSB* with GCase activity and lysosomal function—either through direct interaction (*SCARB2*)^9^, pH regulation (*TMEM175*)^10,11^, or cathepsin B(*CTSB*) in mediating prosaposin cleavage to form saposin C, the lysosomal coactivator of GCase^12,13^. In addition to their mechanistic relevance, these three genes were identified as genome-wide significant loci in large-scale GWAS of PD^3^, further supporting their contribution to disease risk. Together, these genes interact to provide a cohesive framework to dissect lysosomal mechanisms in PD. Their distinct roles are detailed below.

The *TMEM175* gene encodes a proton-selective ion channel located on lysosomal membranes. It mediates the lysosomal H+ leak that balances vacuolar-type H + -ATPase (V-ATPase) activity which helps maintain lysosomal pH homeostasis^11^. Two common coding variants in the *TMEM175* gene, p. Met393Thr (rs34311866) and p. Gln65Pro (rs34884217), show opposite effects on PD susceptibility in several populations^7,14,15^. The GWAS identified p. Met393Thr associated with increased risk (2025 PD GWAS, OR = 1.19)^3,5^ and earlier age of onset of PD^14,16^.

SCARB2, known as lysosomal integral membrane protein-2 (LIMP-2), is an intracellular receptor which shuffles GCase from the endoplasmic reticulum to the lysosome. Variants in this gene may lead to functional and structural lysosomal dysfunction^9^. Two common intronic variants, rs6812193 and rs6825004 (2025 PD GWAS, rs6825004, OR = 0.96; rs6812193, OR = 0.93), have been associated with the risk of PD in several genetic studies^3,5,9,17–19^.

*CTSB* is a cysteine protease, which plays an essential role in lysosomal degradation of α-syn^12^. The *CTSB* locus harbors an intronic variant (rs1293298) which is a common genetic risk factor for PD (2025 PD GWAS, OR = 0.93)^3,5^. Interestingly, this variant also modifies PD risk in *GBA1* carriers, suggesting an interaction between *GBA1* and *CTSB*^6^. This variant lowers the penetrance of *GBA1* mutations, reducing the risk of PD in carriers.

To overcome limitations of prior studies, including insufficient sample sizes for robust statistical inference, ancestry heterogeneity or Eurocentric cohort bias, and fragmented assessments of lysosomal genes contributions, we leveraged large-scale cohorts of genotyping data from GP2 (Global Parkinson’s Genetics Program) and investigated association of common and rare variants in lysosomal related genes (*TMEM175*, *SCARB2*, *CTSB* and *GBA1*) with PD across ten populations.

## Results

### Association analysis of known common risk variants in *TMEM175*, *SCARB2*, and *CTSB* across ten populations

**Fig. 1:**
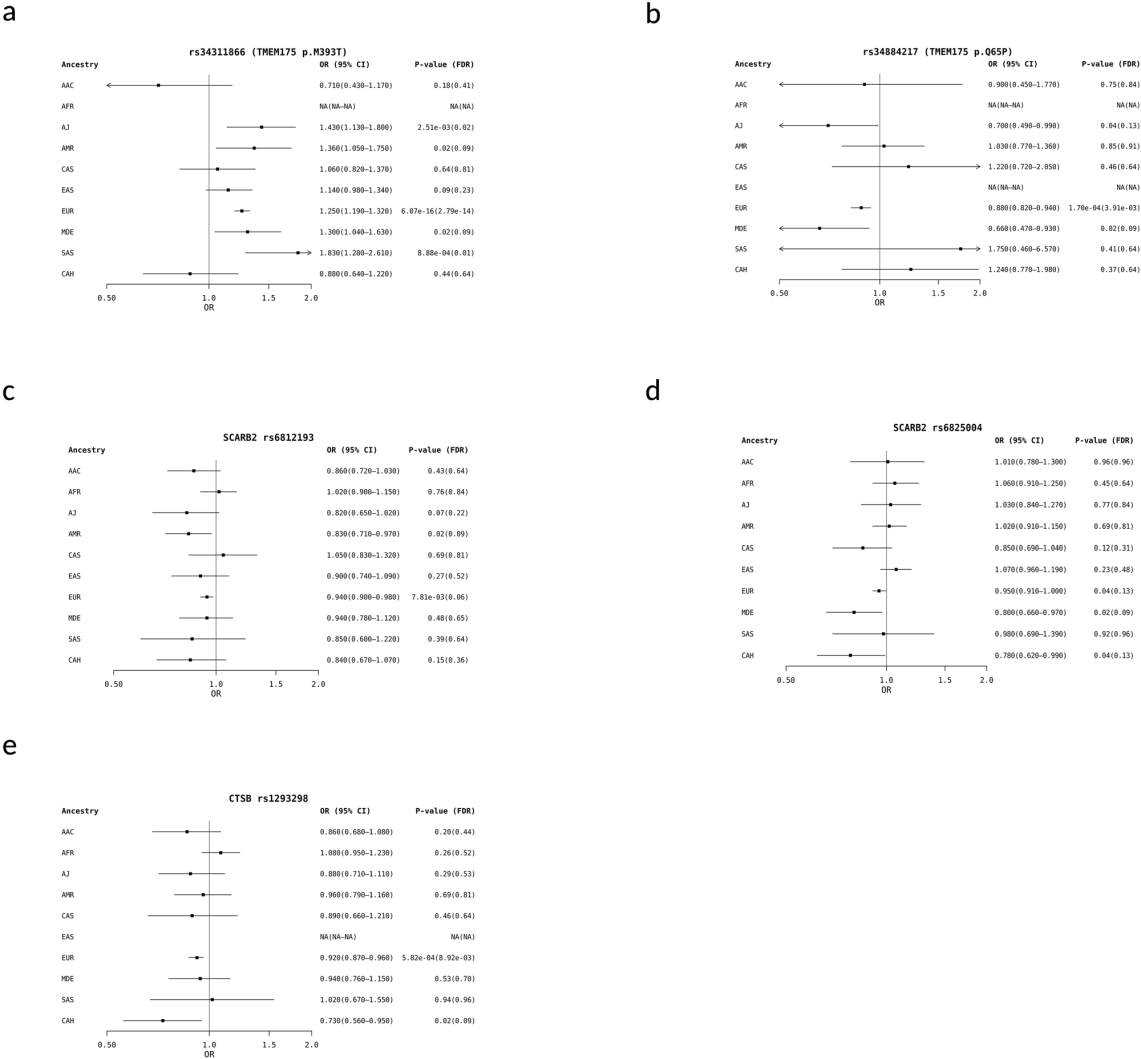
Forest plot illustrating the association between Parkinson’s disease risk and the five studied genetic variants in three lysosomal related genes across ten ancestries. **a** *TMEM175* p.M393T (rs34311866)*,* **b** *TMEM175* p.Q65P (rs34884217), **c** *SCARB2* rs6812193, **d** *SCARB2* rs6825004, and **e** *CTSB* rs1293298*.* P values were corrected for multiple testing across ten ancestries and five genetic variants using the Benjamini–Hochberg method to control the false discovery rate. Abbreviations: AAC African American, AFR African, AJ Ashkenazi Jewish, AMR Latino and indigenous Americas, CAS Central Asian, EAS East Asian, EUR European, MDE Middle Eastern, SAS South Asian, CAH Complex Admixture History.

To validate previously reported GWAS hits, we tested the association of the following variants with PD risk in the GP2-EUR cohort: *TMEM175* (p. Met393Thr and p. Gln65Pro), *SCARB2* (rs6812193 and rs6825004), and *CTSB* (rs1293298). We replicated previous findings that both *TMEM175* variants and *CTSB* variant were significant in the GP2-EUR cohort (Fig. 1a, b, e, Supplementary Table 1a and 1c). However, both variants in *SCARB2*, rs6812193 and rs6825004 did not remain significant after correction for multiple testing (Fig. 1c, d, Supplementary Table 1b).

The known risk factor in *TMEM175*, p. Met393Thr was found to be significantly associated with PD in the GP2-SAS cohort [corrected *P* value = 0.010, OR (95%CI) = 1.83(1.28–2.61)], and GP2-AJ cohort [corrected *P* value = 0.023, OR (95%CI) = 1.43 (1.13–1.80)]. The remaining nominally significant associations did not surpass multiple testing correction (Fig. 1 and Supplementary Table 1a–c). Notably, the *TMEM175* p. Met393Thr had a MAF below 0.01 in the African ancestry and p. Gln65Pro variant in the African and East Asian ancestry and were therefore not analysed.

### Identification of independent signals with conditional analysis across ten populations

The GWAS signal in the *TMEM175* gene, p. Met393Thr was validated as an independent signal in the GP2-EUR cohort (pJ = 6.65e-16) (Table 1; Supplementary Fig. 1a). In the GP2-AJ cohort, rs6599388 (pJ = 2.88e-06) was identified as an additional distinct signal (Table 1; Supplementary Fig. 1b). We found rs6599388 was correlated with the previously reported variant p. Met393Thr (D’ = 0.87, R^2^ = 0.39, *P* < 0.0001) in the GP2-AJ cohort. In the GP2-EAS cohort, another signal rs3755956 was identified as a novel independent signal, showing no correlation with p. Met393Thr (D’ = 0.12, R^2^ = 0.0003, *P* = 0.59) (Table 1; Supplementary Fig. 1c).

**Table 1:** Independent signals in *TMEM175*, *SCARB2* and *CTSB*

*TMEM175*									
Ancestry	variant	A1	A2	MAF (cases)	MAF (controls)	OR (95%CI)	*P* value	pJ	location
EUR	rs34311866 p. Met393Thr	C	T	0.21	0.18	1.25(1.19-1.32)	6.07e-16	6.65e-16	exon
AJ	rs6599388	T	C	0.45	0.54	0.60(0.48-0.74)	2.41e-06	2.88e-06	intron
EAS	rs3755956	T	C	0.094	0.11	0.71(0.60-0.84)	7.87e-05	8.14e-05	intron
*SCARB2*									
EUR	rs11547135	T	C	0.36	0.33	1.11(1.06-1.16)	1.01e-05	1.01e-05	intron
AJ	rs530111925	T	C	0.011	0.022	0.24(0.13-0.47)	3.03e-05	3.22e-05	intron
AMR	rs73828719	G	C	0.083	0.063	0.63(0.50-0.78)	3.55e-05	3.71e-05	intron
*CTSB*									
EUR	rs1293289	A	G	0.27	0.29	0.89(0.85-0.93)	1.13e-06	1.14e-06	intron
AMR	rs73551266	C	T	0.023	0.021	0.48(0.33-0.70)	1.52e-04	1.56e-04	intron

Location of variants are based on GRCh38/hg38. Bonferroni correction was applied for multiple testing to define the significance threshold. The corrected *p*-value thresholds were 4.67e-04 for *TMEM175*, 3.55e-04 for *SCARB2*, and 4.31e-04 for *CTSB*.

*AJ* Ashkenazi Jewish, *AMR* Latino and indigenous Americas, *EAS* East Asian, *EUR* European, *A1* alternative allele, *A2* reference allele, *MAF* minor allele frequency, *OR* odds ratio, *P value,* *p*-value from the original association analysis, *pJ* *p*-value from a joint analysis of all the selected variants.

We identified one independent signal, rs11547135(pJ = 1.01e-05), in *SCARB2* in the GP2-EUR cohort (Table 1; Supplementary Fig. 2a). rs11547135 was not correlated with previously reported variants rs6812193(D’ = 0.43, R^2^ = 0.056, *P* < 0.0001) and rs6825004(D’ = 0.27, R^2^ = 0.073, *P* < 0.0001). Additionally, colocalization analysis showed that rs11547135 was strongly associated with SCARB2 expression (posterior probability for a shared causal variant, H4 > 0.99; Supplementary Table 2). Consistent findings were observed across the eQTLGen and GTEx datasets, indicating that the effect allele T was associated with upregulated gene expression in both blood and brain tissues. One additional novel independent genetic variants in *SCARB2*, rs530111925(pJ = 3.22e-05) was identified in the GP2-AJ cohort(Table 1; Supplementary Fig. 2b) and this variant was not correlated with rs6812193(D’ = 0.31, R^2^ = 0.0027, *P* < 0.0001) and rs6825004(D’ = 0.14, R^2^ = 0.00035, *P* = 0.12). In the GP2-AMR cohort, one additional distinct signal, rs73828719 (pJ = 3.71e-05) was correlated with rs6812193 (D’ = 0.87, R^2^ = 0.26, *P* < 0.0001) but not correlated with rs6825004 (D’ = 0.19, R^2^ = 0.0028, *P* < 0.0001)(Table 1; Supplementary Fig. 2c).

We identified one conditionally distinct association signal in *CTSB*, rs1293289(pJ = 1.14e-06), in the GP2-EUR cohort, but rs1293289 was correlated with the known PD GWAS hit rs1293298 (D’ = 0.97, R^2^ = 0.80, *P* < 0.0001) (Table 1; Supplementary Fig. 3a). In the GP2-AMR cohort, one independent novel genetic variants in *CTSB*, rs73551266(pJ = 1.56e-04) was identified (Table 1; Supplementary Fig. 3b) and rs73551266 was independent of rs1293298(D’ = 0.0038, R^2^ = 0.00, *P* = 0.96).

### Burden analysis with GP2 neurobooster array

To test if an aggregate burden of rare variants in *TMEM175*, *SCARB2* and *CTSB* contributes to the risk of PD, SKAT-O analysis was conducted within the GP2 Neurobooster array data across ten populations (Table 2).

**Table 2:** Skat-O analysis of *TMEM175, SCARB2* and *CTSB*

*TMEM175*						*SCARB2*				*CTSB*			
Ancestry	Variants	Num Var	rho	*P*-value	FDR corrected *P*-value	NumVar	rho	*P* value	FDR corrected *P*-value	NumVar	rho	*P* value	FDR corrected *P*-value
AAC	exonic	59	0	0.63	0.96	21	0	1.87e-03*	0.11	28	0	0.75	0.96
	Nonsyn and LoF	38	0	0.52	0.96	12	0	0.050	0.59	15	0	0.28	0.96
AFR	exonic	76	1	0.74	0.96	19	1	0.78	0.96	46	0	0.83	0.96
	Nonsyn and LoF	47	1	0.74	0.96	10	1	0.28	0.96	25	0.30	0.15	0.89
AJ	exonic	24	0	0.75	0.96	5	0	0.021*	0.35	13	1	0.73	0.96
	Nonsyn and LoF	15	1	0.58	0.96	4	NA	NA	NA	11	1	0.88	0.96
AMR	exonic	72	0	0.62	0.96	18	0	0.85	0.96	39	0	0.75	0.96
	Nonsyn and LoF	42	1	0.83	0.96	14	0	0.50	0.96	27	0	0.47	0.96
CAS	exonic	39	1	0.18	0.96	15	0	0.13	0.89	18	1	0.86	0.96
	Nonsyn and LoF	26	0	0.26	0.96	10	0.2	0.091	0.77	12	1	0.76	0.96
EAS	exonic	52	0	0.86	0.96	17	0	0.024*	0.35	24	1	1	1
	Nonsyn and LoF	34	0	0.40	0.96	12	0	0.019*	0.35	17	1	0.54	0.96
EUR	exonic	128	0	0.41	0.96	43	0	0.52	0.96	93	0	0.57	0.96
	Nonsyn and LoF	69	1	0.86	0.96	27	0	0.54	0.96	59	1	0.56	0.96
MDE	exonic	47	0	0.42	0.96	21	1	1	1	24	1	0.40	0.96
	Nonsyn and LoF	24	0	0.066	0.65	15	1	1	1	15	1	0.57	0.96
SAS	exonic	25	0	0.52	0.96	10	0	0.29	0.96	19	1	0.42	0.96
	Nonsyn and LoF	18	1	0.55	0.96	7	0.8	0.15	0.89	12	0	0.64	0.96
CAH	exonic	47	1	0.64	0.96	16	1	1	1	28	0	0.73	0.96
	Nonsyn and LoF	30	0	0.43	0.96	9	1	1	1	14	1	0.82	0.96

*P* values were corrected for multiple testing across ten ancestries, three genes and two variant categories using the Benjamini–Hochberg method to control the false discovery rate.

*AAC* African American, *AFR* African, *AJ* Ashkenazi Jewish, *AMR* Latino and indigenous Americas, *CAS* Central Asian, *EAS* East Asian, *EUR* European, *MDE* Middle Eastern, *SAS* South Asian, *CAH* Complex Admixture History, *Nonsyn* non-synonymous variants, *LoF* and Loss of function, *NA* not available, *NumVar* number of variants.

* *p* value < 0.05.

No significant associations were detected for rare exonic and non-synonymous and LoF variants in *TMEM175* and *CTSB* across ten GP2 cohorts. In addition, we conducted SKAT-O analyses including the known GWAS hits *TMEM175* p. Met393Thr and p. Gln65Pro as covariates, and the results were summarized in Supplementary Table 3a.

Similarly, a SKAT-O analysis for *SCARB2* was run to detect a potential genetic burden in PD cases versus controls (Table 2). Subsequently, a SKAT test was performed to further validate SKAT-O results according to the rho value^20^ (Supplementary Table 3b). However, no associations survived multiple-testing correction (Table 2). To determine which single variants may be driving the observed association with PD risk, a single-variant-based test was conducted. Nominal associations were detected in the GP2-AAC, GP2-AJ, and GP2-EAS cohorts; however, none remained significant after multiple-testing correction (Table 3).

**Table 3:** Significant signals with single variant burden analysis (*SCARB2*)

Ancestry	Variant	A1	A2	MAF_ cases	MAF_controls	N_cases	N_controls	effect	SE	Direction	*P*-value	FDR corrected *P*-value	MAF (GnomAD)
AAC	p.Val149Met	T	C	1.36e-03	1.32e-03	306	712	11.52	4.43	+	9.29e-03*	0.19	2.15e-03 (global)
AJ	p. Met159Val	C	T	5.06e-03	0.011	1209	219	−5.25	2.27	-	0.021*	0.36	0.013
EAS	p. Asp194Asn	T	C	6.32e-04	1.16e-03	2260	1505	−4.31	1.78	-	0.015*	0.27	2.10e-03
	p. Ile144Leu	A	T	4.89e-03	3.10e-03	2241	1492	2.05	0.91	+	0.024*	0.34	8.30e-03

*P* values were adjusted for multiple testing across ten ancestries, correcting for all single-variant tests within the SCARB2 locus using the Benjamini–Hochberg method to control the false discovery rate. Global refers to the worldwide MAF reported in gnomAD, as ancestry specific MAF for the African American cohort was not available.

*AAC* African American, *AJ* Ashkenazi Jewish, *EAS* East Asian, p. Val149Met(rs147159813), p. Met159Val(rs143655258), p. Asp194Asn(rs773017713), p. Ile144Leu(rs117600063), *A1* alternative allele, *A2* reference allele, *MAF* minor allele frequency, *SE* stanadard eror, + means risk, - means protective.

* *p* value < 0.05.

## Discussion

We show that previous GWAS hits of three lysosomal related genes (*TMEM175*, *SCARB2* and *CTSB*) were successfully replicated using large scale genotyping array data. All previously detected variants were significant in the cohort of European ancestry and in some additional ancestry cohorts. The GCTA-COJO analysis was performed across all ten cohorts to identify novel independent signals in these three lysosome-related genes. Significant associations were observed in GP2-EUR, GP2-AJ, GP2-EAS, and GP2-AMR cohorts. Furthermore, SKAT-O analysis revealed genetic burdens for *SCARB2*, showing nominal significant associations in distinct cohorts. Collapsed burden tests and SKAT tests were further used to validate these findings. Moreover, we detected single rare variants contributing to PD risk through single-variant-based tests.

The missense variant p. Met393Thr in the *TMEM175* gene was the most strongly associated signal in the region. The association reached genome-wide significance and *TMEM175* p. Met393Thr was identified as a single independent risk factor of PD in the European population, aligning with previous reports^3^. In addition, it was associated with PD in Ashkenazi Jewish and South Asian populations, which could be possibly due to a haplotype block including p. Met393Thr variant in these other populations. Another common variant rs34884217 p. Gln65Pro, as a secondary signal in the same locus, was found to be associated with reduced PD risk in a previous study^21^. In this study, the frequency of the protective allele was also significantly higher in the controls than in cases in European population. Notably, due to the MAF threshold of 0.01 for common variants, *TMEM175* p. Met393Thr and p. Gln65Pro in the African, and *TMEM175* p. Gln65Pro in the East Asian populations, were not included in the analysis in these populations.

We went further to identify independent signals in *TMEM175* using GCTA-COJO analysis in other populations. Genotypes at rs6599388 in the *TMEM175* gene were detected as an additional distinct intronic variant for PD risk with GCTA-COJO in the Ashkenazi Jewish population. Of note, *TMEM175* rs6599388 was correlated with p. Met393Thr. It was also previously reported to be associated with PD in GWA studies in the European population (2011 PD GWAS, rs6599388, odds ratio [OR] = 1.16)^22^. In the East Asian population, conditional analysis identified rs3755956 as a novel, independent association signal at the *TMEM175* locus.

No statistically significant associations of rare variants in the *TMEM175* gene were found for any ancestral populations. A previous study did not show significant results in 2657 patients and 3647 controls in a *TMEM175* burden analysis^23^. Our finding was consistent with this previous study, supported by sufficient statistical power, suggesting that a cumulative burden of rare variants in the *TMEM175* gene does not contribute significantly to PD risk.

Prior studies in European-ancestry cohorts reported associations at rs6812193 and rs6825004 in *SCARB2* with PD risk^3,9,18,19^. However, after correcting for multiple testing across variants and ancestries, none of the associations remained significant, neither in Europeans nor in any other population.

Besides this, we identified one novel independent non-coding common genetic variant (rs11547135) in the European population. Colocalization analysis showed rs11547135 was strongly associated with SCARB2 expression in blood (eQTLGen) and brain (GTEx) tissue, with consistent positive effect directions. This concordance suggested a shared regulatory mechanism influencing gene expression across tissues, supporting its role as a potential functional variant contributing to PD risk. Further functional studies are warranted to validate this regulatory mechanism and to elucidate its contribution to the molecular pathways in PD pathogenesis. In the Ashkenazi Jewish population, rs530111925 at the *SCARB2* locus was identified as a novel, independent variant. Excessive genetic burden of *SCARB2* rare variants was not replicated in the European population. Previous studies had shown a possible association between *SCARB2* rare variants and PD risk with SKAT-O test^8,23^. This may be attributed to genetic and demographic differences among populations from different regions or lack of statistical power. Interestingly, *SCARB2* rare variants in exonic regions showed nominal associations with PD risk in three other populations: the African American, the Ashkenazi Jewish, and the East Asian cohorts. We found one novel single genetic variant, p. Val149Met, was associated with increased PD risk in the African American population. In the Ashkenazi Jewish cohort, single-variant-based tests indicated that p. Met159Val might have a protective effect on PD. In the East Asian population, we identified two nominally significant missense variants, p. Asp194Asn and p. Ile144Leu, which exhibited discordant effects on PD. None of these significant single genetic variants has previously been reported in the context of PD risk. Furthermore, rare variant analysis of *SCARB2* revealed mixed effect directions, with a balanced effect magnitude across the two directions. In such scenarios, the aggregate effect may appear null, despite the presence of biologically meaningful associations. The bidirectional effects have also been observed in genes like *TMEM175* and *CTSB*, where opposing variant effects can obscure the cumulative genetic burden on disease risk. These findings underscore a key limitation of conventional burden analyses that rely on the assumption of effect directionality. On the other hand, understanding such discordance was crucial as it suggested that targeting a single specific pathogenic variant—rather than the gene as a whole—may be a more effective strategy in clinical interventions.

We found *CTSB* rs1293298 as a significant risk factor in the European population. Of note, *CTSB* rs1293298 in East Asians was excluded from the common variant analyses due to MAF < 0.01. In the European cohort, we identified a conditionally distinct common intronic variant (rs1293289), linked to the known GWAS hit *CTSB* rs1293298. In addition, *CTSB* rs73551266 was detected as a novel independent variant in the Latino and indigenous Americas population.

A recent study observed a nominal association between *CTSB* rare variants and PD risk in a single Ashkenazi Jewish cohort^24^. However, we did not replicate this result in large European cohort (24,208 cases, 9662 controls) and any other populations. In contrast to the previous study, we excluded carriers of *GBA1* variants from all cohorts to minimize potential bias, which may partly explain the discrepancy. Our findings further support the limitation of rare variant burden analysis, particularly in genes like *TMEM175* and *CTSB*, where both risk and protective variants may co-exist. Besides, future studies should focus on larger sample size in other populations to increase robustness.

The associations of known GWAS hits with PD in other populations were observed although not reaching genome wide significance. This discrepancy may reflect distinct LD patterns, differences in haplotype structures among populations or limited statistical power due to smaller cohort size. Future haplotype-resolved analyses (e.g., phased whole-genome sequencing or ancestry-stratified fine-mapping) could clarify whether these associations arise from shared causal variants present on divergent haplotypes or population-specific functional variants embedded within risk haplotypes. On the other hand, our study illustrates heterogeneity in genetic associations across populations in PD. These combined analyses emphasize these three lysosomal related genes (*TMEM175*, *SCARB2* and *CTSB*) driving PD risk via both common variants and rare variants. Future research should prioritize detailed functional characterization of these novel variants and their potential patho-mechanism in diverse populations to uncover their roles, facilitating improved understanding and potential development of targeted interventions for PD.

The strength of this study is the analysis of a large-scale case-control cohort from the GP2 Neurobooster array with a population of European ancestry and nine additional populations. The observed replication may, in part, be influenced by partial sample overlap with the 2019 PD GWAS cohort. Notably, 7 out of the 126 cohorts included in our study were included in the previous Nalls’s PD GWAS. It is not possible to identify exactly which samples overlap, because the genotyping arrays are so different that kinship analysis does not identify related/duplicated samples. In addition, as *GBA1* lies adjacent to its highly homologous pseudogene *GBAP1*, accurate variant calling in short-read data such as the Neurobooster array can be challenging. Moreover, the array does not capture all rare variants in *GBA1* gene, which may result in some carriers not being detected. These factors should be taken into account when interpreting the findings. Although conditional analysis identified independent signals in distinct populations, the strong linkage disequilibrium (LD) between variants and the lack of replication in non-European cohorts indicated potential population specificity. Therefore, these associations should be interpreted with caution, and further large-scale studies across diverse ancestries are essential to confirm their robustness and generalizability. In the single-variant analyses, the very small number of carriers for ultra-rare variants can yield unstable effect-size estimates and spuriously small *p*-values under the score test framework. These signals should be viewed cautiously until replicated in larger cohorts, and further methodological development for ultra-rare variants will be warranted.

Importantly, our study reinforces the association between lysosomal genes and PD risk in the European population. The number of non-European individuals will need to be increased in future studies, being clearly smaller compared to the European cohort. PD is a complex disorder with many genetic risk factors at multiple loci contributing to disease risk. Therefore, the novel variants reported in this study should be further investigated in terms of functional effects on PD pathogenesis in the future.

## Methods

### Subjects

To validate the association between common variants in lysosomal genes from the reported GWA study of PD^3^, large-scale Neurobooster genotyping imputed data obtained from GP2 release 10 (https://gp2.org/the-components-of-gp2s-10th-data-release/) was analysed, which contains 33,733 cases and 18,703 controls in ten ancestries: GP2-African American (GP2-AAC: 369 cases, 756 controls), GP2-African (GP2-AFR: 1147 cases, 2169 controls), GP2-Ashkenazi Jewish (GP2-AJ: 1285 cases, 408 controls), GP2-Latino and indigenous Americas (GP2-AMR: 1917 cases, 1402 controls), GP2-Central Asian (GP2-CAS: 734 cases, 578 controls), GP2-East Asian (GP2-EAS: 2377 cases, 2598 controls), GP2-European (GP2-EUR: 24,208 cases, 9662 controls), GP2-Middle Eastern (GP2-MDE: 724 cases, 551 controls), GP2-South Asian (GP2-SAS: 309 cases, 261 controls), GP2-Complex Admixture History (GP2-CAH: 663 cases, 318 controls). Populations with less than 100 individuals (i.e. Finnish) were excluded. Given the strong influence of *GBA1* on lysosomal biology and its close interactions with other lysosomal related genes, individuals carrying established *GBA1* risk-associated variants were removed across all ancestries to avoid inflating the apparent contribution of other lysosomal related genes and to assess their independent effects. This approach referred to a previous study^25^ and minimize potential bias from co-occurrence with *GBA1* variants. The number of individuals removed was provided in Supplementary Table 4. Quality control analyses at a sample and variant level for this GP2 dataset were described elsewhere (https://github.com/GP2code/GenoTools)^26^. At the sample level, quality control included call rate outliers(--mind, 0.05), biologic sex mismatches (--check-sex, default cutoffs: 0.25 ≤ F ≤ 0.75), relatedness check (--grm-cutoff, in GCTA; Usually set at 0.125 to remove first cousins or more related individuals), and heterozygosity rate outliers (--het, default range: −0.15 ≤ F ≤ 0.15). At the variant level, quality control included missingness by case control (--test-missing, using *P* > 1e-04), missing by haplotype (--test-mishap, using *P* > 1e-04), Hardy–Weinberg equilibrium (--filter-controls --hwe, using *P* > 1e-04) and variant missingness (--geno, 0.05). Cases and controls with missing covariates (including gender and age) were removed.

### Gene region extraction and variants annotation

Regions of each gene were extracted with PLINK 2.0 based on the gene locations according to the Ensembl genome browser (https://www.ensembl.org/Homo_sapiens/), including 50 kb downstream and upstream of the genes. Variants were annotated using ANNOVAR^27^, and variants were defined as (1) exonic, (2) non-synonymous and loss of function (LoF). LoF included stopgain, stoploss and frameshift variants.

### Statistical analysis

The number of principal components was chosen by the scree plot of explained variance (Supplementary Fig. 4), which showed a clear elbow after PC5; accordingly, PC1–PC5 were included as covariates.

Association analyses were performed with PLINK 2.0^28^, adjusted by gender, age and the first five genetic principal components (PCs) in each ancestry.

Conditional and joint (COJO) analysis was conducted to determine if there were independent signals per ancestry with genome-wide complex trait analysis (GCTA) software version 1.94.1^29,30^. To identify gene-wide significant signals in the absence of genome-wide significance (*P* < 5 × 10⁻⁸), we established gene-specific significant thresholds through linkage disequilibrium (LD) pruning followed by Bonferroni correction. LD pruning was performed to define a *p*-value threshold for the COJO analysis in GP2 NeuroBooster array (parameters: --indep-pairwise 50 50 0.5). We identified 107 independent variants in *TMEM175*, 141 variants in *SCARB2* and 116 variants in *CTSB*. Therefore, the thresholds for *P* values which were used in conditional analysis were: 4.67e-4 (0.05/107, *TMEM175*), 3.55e-4 (0.05/141, *SCARB2*), 4.31e-4 (0.05/116, *CTSB*). In addition, LDpair tool (https://ldlink.nih.gov/?tab=ldpair) was used to validate if these signals were independent of known GWAS hits. Regional plots were generated with the online tool LocusZoom.js (https://statgen.github.io/localzoom/)^31^.

Colocalization analysis was performed to assess whether the genetic variant colocalized with expression quantitative trait locus(eQTL) signals using eQTLGen public dataset(https://www.eqtlgen.org/cis-eqtls.html), following previously described pipelines^32^(https://rhreynolds.github.io/RBD-GWAS-analysis/, https://github.com/manuelatan/PD-survival-GWAS/). The eQTLGen consortium provides eQTL data representing gene expression in whole blood. To further assess tissue specificity and consistency of effect direction, the GTEx public dataset (https://www.gtexportal.org/home/) was additionally examined to validate the association in brain tissue.

GP2 NeuroBooster array dataset was used to assess whether *TMEM175*, *SCARB2*, and *CTSB* have an excessive genetic burden on PD. Gene-based burden tests were performed using RVTESTS^33^ to assess the cumulative effects of rare variants on the risk for PD. We defined rare variants as MAF ≤ 1% (--freqUpper 0.01) and applied this same threshold to both gene-based and single-variant-based tests. We used the default Beta (1,25) kernel weights, which up-weight rare variants; and all other parameters were set by default. Analyses were performed twice for each gene, once for all exonic variants and once for the stricter subset of non-synonymous +LoF variants. Furthermore, single-variant-based tests were conducted with RVTESTS (https://zhanxw.github.io/rvtests/#single-variant-tests-1) using a score test under a logistic regression model (--single score) for the case–control phenotype to find single statistically significant variants which have an impact on PD risk^33^. All burden analyses were adjusted for age, gender and PC1-PC5.

To account for multiple testing, raw *p*-values were adjusted using the Benjamini–Hochberg (BH) method to control false discovery rate (FDR). For the association analysis of known common genetic variant, corrections were applied across ten ancestries and five genetic variants. For the SKAT-O tests, *p*-values were adjusted across ten ancestries, three genes, and two variant categories. For the single-variant analyses, corrections were applied to all single-variant tests across ten ancestries using the same method.

All analyses were performed using R statistical language, Python and Linux programming language on Terra (https://terra.bio/) and Verily workbench (https://workbench.verily.com/).

**Fig. 2:**
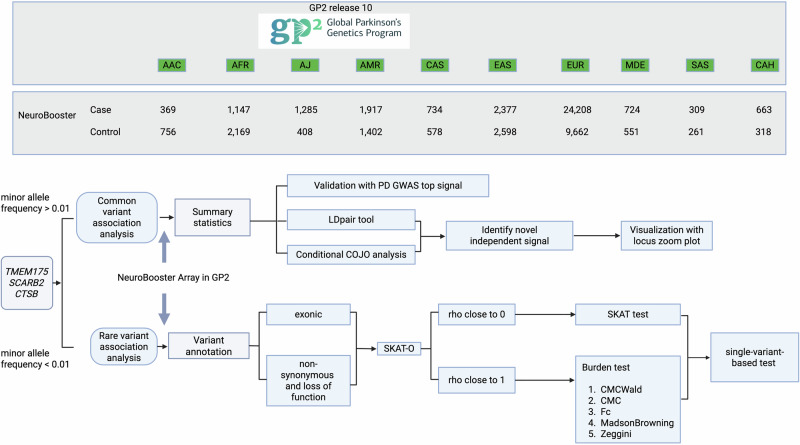
General overview of analysis.

Our overall analytic approach is illustrated in Fig. 2.

### Ethics and consent

Written informed consent was obtained from all participants (or their parent/legal guardian in the case of individuals under 16 years of age). For multi-centre studies, each collaborating site secured approval from their local ethics committee, and consent procedures were reviewed by the Operations and Compliance Working Group (OCWG) of GP2 to ensure compliance with international standards and local data-sharing regulations. A list of all participating sites can be found on the GP2 website (https://gp2.org/cohort-dashboard-advanced/). Detailed information on ethics approvals and consent procedures is available on the GP2 website (https://gp2.org/resources/consent-guidelines/).

## Supplementary information

supplementary material

## Data Availability

Data used in the preparation of this article were obtained from the Global Parkinson’s Genetics Program (GP2; https://gp2.org). Specifically, we used Tier 2 data from GP2 release 10 (10.5281/zenodo.15748014). GP2 data are available on AMP PD (https://amp-pd.org).

## Code availability

All code generated for this article, and the identifiers for all software programs and packages used, are available on GitHub (https://github.com/GP2code/TMEM175_SCARB2_CTSB_PDrisk) and were given a persistent identifier via Zenodo (10.5281/zenodo.15799510).
