# Author Correction: *TMEM175*, *SCARB2* and *CTSB* associations with Parkinson’s disease risk across populations

**DOI:** 10.1038/s41531-026-01351-6

**Published:** 2026-04-13

**Authors:** Wenhua Sun, Claudia Schulte, Thomas Gasser, Manuela Tan, the Global Parkinson’s Genetic Program (GP2), Lasse Pihlstrøm, Pedro Chana, Yeajin Song, Sara Bandres-Ciga, Cornelis Blauwendraat, Andrew Singleton, Mike A. Nalls, Hampton Leonard, Mie Rizig, Hirotaka Iwaki, Carlos Rieder, Ignacio F. Mata, Njideka Okubadejo, Emilia M. Gatto, Marcelo Kauffman, Claire E. Shepherd, Samson Khachatryan, Zaruhi Tavadyan, Julie Hunter, Kishore Kumar, Melina Ellis, Miguel E. Rentería, Sulev Koks, Alexander Zimprich, Vitor Tumas, Sarah Camargos, Edward A. Fon, Ted Fon, Oury Monchi, Benjamin Pizarro Galleguillos, Patricio Olguin, Marcelo Miranda, Maria Leonor Bustamante, Beisha Tang, Huifang Shang, Jifeng Guo, Piu Chan, Wei Luo, Gonzalo Arboleda, Jorge Orozco, Marlene Jimenez del Rio, Alvaro Hernandez, Mohamed Salama, Walaa A. Kamel, Yared Z. Zewde, Alexis Brice, Jean-Christophe Corvol, Ana Westenberger, Christine Klein, Eva-Juliane Vollstedt, Harutyun Madoev, Joanne Trinh, Johanna Junker, Katja Lohmann, Anastasia Illarionova, Brit Mollenhauer, Franziska Hopfner, Günter Höglinger, Lara M. Lange, Manu Sharma, Sergio Groppa, Zih-Hua Fang, Albert Akpalu, Georgia Xiromerisiou, Georgios Hadjigeorgiou, Efthymios Dardiotis, Ioannis Dagklis, Ioannis Tarnanas, Leonidas Stefanis, Maria Stamelou, Alex Medina, Germaine Hiu-Fai Chan, Nelson Yuk-Fai Cheung, Nancy Ip, Phillip Chan, Xiaopu Zhou, Asha Kishore, K. P. Divya, Pramod Pal, Prashanth Lingappa Kukkle, Roopa Rajan, Rupam Borgohain, Mehri Salari, Andrea Quattrone, Enza Maria Valente, Micol Avenali, Lucilla Parnetti, Tommaso Schirinzi, Manabu Funayama, Nobutaka Hattori, Tomotaka Shiraishi, Altynay Karimova, Gulnaz Kaishibayeva, Cholpon Shambetova, Rejko Krüger, Ai Huey Tan, Azlina Ahmad-Annuar, Shen-Yang Lim, Yi Wen Tay, Mohamed Ibrahim Norlinah, Shahrul Azmin, Nor Azian Abdul Murad, Daniel Martinez-Ramirez, Mayela Rodriguez-Violante, Paula Reyes-Pérez, Bayasgalan Tserensodnom, Rajeev Ojha, Tim J. Anderson, Toni L. Pitcher, Oluwadamilola Ojo, Jan O. Aasly, Shoaib Ur-Rehman, Mario Cornejo-Olivas, Maria Leila Doquenia, Raymond Rosales, Angel Vinuela, Elena Iakovenko, Bashayer Al Mubarak, Muhammad Umair, Eng-King Tan, Ferzana Amod, Jonathan Carr, Soraya Bardien, Beomseok Jeon, Yun Joong Kim, Esther Cubo, Ignacio Alvarez, Janet Hoenicka, Katrin Beyer, Pau Pastor, Sarah El-Sadig, Christiane Zweier, Paul Krack, Chin-Hsien Lin, Ruey-Meei Wu, Hsiu-Chuan Wu, Yih-Ru Wu, Pin-Jui Kung, Serena Wu, Rim Amouri, Samia Ben Sassi, A. Nazl Başak, Özgür Öztop Çakmak, Sibel Ertan, Gencer Genc, Alejandro Martínez-Carrasco, Anette Schrag, Anthony Schapira, Eleanor J. Stafford, Henry Houlden, Huw R. Morris, John Hardy, Nicholas Wood, Olaitan Okunoye, Rauan Kaiyrzhanov, Rimona Weil, Simona Jasaitye, Vida Obese, Camille Carroll, Claire Bale, Donald Grosset, Kin Y. Mok, Nigel Williams, Patrick A. Lewis, Seth Love, Simon Stott, Alberto Espay, Luca Marsili, Alyssa O’Grady, Bernadette Siddiqi, Bradford Casey, Brian Fiske, Charisse Comart, Justin C. Solle, Kaileigh Murphy, Maggie Kuhl, Naomi Louie, Sohini Chowdhury, Todd Sherer, Andrew K. Sobering, Cabell Jonas, Carlos Cruchaga, Caroline B. Pantazis, Claire Wegel, Deborah Hall, Ejaz Shamim, Jared Williamson, Ekemini Riley, Sonya Dumanis, Geidy E. Serrano, Thomas Beach, Honglei Chen, Ignacio Juan Keller Sarmiento, Niccolò E. Mencacci, Steven Lubbe, Joseph Jankovic, Miguel Inca-Martinez, Joshua Shulman, Karen Nuytemans, Karl Kieburtz, Katerina Markopoulou, Kenneth Marek, Lana M. Chahine, Lauren Ruffrage, Marissa Dean, Lisa Shulman, Roger Albin, Roy Alcalay, Ruth Walker, Tao Xie, Tatiana Foroud, Duan Nguyen, Toan Nguyen, Masharip Atadzhanov

**Affiliations:** 1 Department of Neurodegenerative Diseases, Hertie Institute for Clinical Brain Research, University of Tuebingen and German Center for Neurodegenerative Diseases (DZNE), Tuebingen, Germany; 2 Department of Neurology, Oslo University Hospital, Oslo, Norway; 3 Centro de Trastornos del Movimiento (CETRAM), Santiago, Chile; 4 Centre for Alzheimer’s and Related Dementias. National Institute on Aging, National Institutes of Health, Bethesda, MD USA; 5 Laboratory of Neurogenetics, National Institute on Aging, National Institutes of Health, Bethesda, MD USA; 6 DataTecnica LLC, Washington, DC USA; 7 Department of Neuromuscular Diseases, UCL Queen Square Institute of Neurology, London, UK; 8 Preventive Neurology Unit, Wolfson Institute of Population Health, Queen Mary University of London, London, UK; 9 Department of Anatomy, College of Medicine, University of Lagos, Lagos, Nigeria; 10 Serviço de Neurologia, Hospital de Clínicas de Porto Alegre, Porto Alegre, Brazil; 11 Universidade Federal do Rio Grande do Sul / Hospital de Clínicas de Porto Alegre, Porto Alegre, Brazil; 12 Genomic Medicine, Lerner Research Institute, Cleveland Clinic Foundation, Cleveland, OH USA; 13 Department of Medicine, College of Medicine, University of Lagos, Lagos, Nigeria; 14 Sanatorio de la Trinidad Mitre-INEBA, Buenos Aires, Argentina; 15 Hospital JM Ramos Mejia, Buenos Aires, Argentina; 16 Neuroscience Research Australia, Sydney, NSW Australia; 17 Somnus Neurology Clinic, Yerevan, Armenia; 18 ANZAC Research Institute, Concord, NSW Australia; 19 Garvan Institute of Medical Research and Concord Repatriation General Hospital, Darlinghurst, NSW Australia; 20 Concord Hospital, Concord, NSW Australia; 21 QIMR Berghofer Medical Research Institute, Herston, QLD Australia; 22 Murdoch University, Perth, WA Australia; 23 Medical University of Vienna, Vienna, Austria; 24 University of São Paulo, São Paulo, Brazil; 25 Universidade Federal de Minas Gerais, Belo Horizonte, Brazil; 26 McGill University, Montreal, QC Canada; 27 Universidad de Chile, Santiago, Chile; 28 Fundación Diagnosis, Santiago, Chile; 29 Faculty of Medicine, Universidad de Chile, Santiago, Chile; 30 CETRAM, Santiago, Chile; 31 Central South University, Changsha, China; 32 West China Hospital, Sichuan University, Chengdu, China; 33 Xiangya Hospital, Central South University, Changsha, China; 34 Capital Medical University, Beijing, China; 35 Zhejiang University, Hangzhou, China; 36 Universidad Nacional de Colombia, Bogotá, Colombia; 37 Fundación Valle del Lili, Santiago de Cali, Colombia; 38 University of Antioquia, Medellín, Colombia; 39 University of Costa Rica, San José, Costa Rica; 40 The American University in Cairo, Cairo, Egypt; 41 Beni-Suef University, Beni Suef, Egypt; 42 Addis Ababa University, Addis Ababa, Ethiopia; 43 Paris Brain Institute, Paris, France; 44 Sorbonne Université, Paris, France; 45 University of Lübeck, Lübeck, Germany; 46 Deutsches Zentrum für Neurodegenerative Erkrankungen (DZNE), Göttingen, Germany; 47 University Medical Center Göttingen, Göttingen, Germany; 48 University Hospital LMU Munich, Munich, Germany; 49 Hannover Medical School, Hannover, Germany; 50 University of Lübeck and University Medical Center Schleswig-Holstein, Lübeck, Germany; 51 University of Tübingen, Tübingen, Germany; 52 The German Center for Neurodegenerative Diseases (DZNE), Göttingen, Germany; 53 University of Ghana Medical School, Accra, Ghana; 54 University of Thessaly, Larissa, Greece; 55 Aristotle University of Thessaloniki, Thessaloniki, Greece; 56 Ionian University, Corfu, Greece; 57 Biomedical Research Foundation of the Academy of Athens, Athens, Greece; 58 Diagnostic and Therapeutic Centre HYGEIA Hospital, Marousi, Greece; 59 Hospital San Felipe, Tegucigalpa, Honduras; 60 Queen Elizabeth Hospital, Kowloon, Hong Kong; 61 The Hong Kong University of Science and Technology, Kowloon, Hong Kong; 62 Aster Medcity, Kochi, India; 63 Sree Chitra Tirunal Institute for Medical Sciences and Technology, Thiruvananthapuram, India; 64 National Institute of Mental Health & Neurosciences (NIMHANS), Bengaluru, India; 65 Manipal Hospital, Delhi, India; 66 All India Institute of Medical Sciences, Delhi, India; 67 Nizam’s Institute of Medical Sciences, Hyderabad, India; 68 Shahid Beheshti University of Medical Sciences, Tehran, Iran; 69 Magna Græcia University of Catanzaro, Catanzaro, Italy; 70 University of Pavia, Pavia, Italy; 71 University of Perugia, Perugia, Italy; 72 University of Rome Tor Vergata, Rome, Italy; 73 Juntendo University, Tokyo, Japan; 74 Juntendo University Faculty of Medicine, Tokyo, Japan; 75 Jikei University School of Medicine, Tokyo, Japan; 76 Institute of Neurology and Neurorehabilitation, Almaty, Kazakhstan; 77 Kyrgyz State Medical Academy, Bishkek, Kyrgyzstan; 78 Luxembourg Centre for Systems Biomedicine, University of Luxembourg, Belvaux, Luxembourg; 79 University of Malaya, Kuala Lumpur, Malaysia; 80 Universiti Kebangsaan Malaysia, Kuala Lumpur, Malaysia; 81 UKM Medical Molecular Biology Institute (UMBI), Kuala Lumpur, Malaysia; 82 Universiti Kebangsaan Malaysia Medical Centre, Kuala Lumpur, Malaysia; 83 Tecnológico de Monterrey, Monterrey, Mexico; 84 Instituto Nacional de Neurología y Neurocirugía, Mexico City, Mexico; 85 Universidad Nacional Autónoma de México, Mexico City, Mexico; 86 Mongolian National University of Medical Sciences, Ulaanbaatar, Mongolia; 87 Tribhuvan University, Kirtipur, Nepal; 88 University of Otago, Christchurch, New Zealand; 89 University of Otago, Dunedin, New Zealand; 90 College of Medicine, University of Lagos, Lagos, Nigeria; 91 Norwegian University of Science and Technology, Trondheim, Norway; 92 University of Science and Technology Bannu, Khyber Pakhtunkhwa, Pakistan; 93 Instituto Nacional de Ciencias Neurológicas, Lima, Peru; 94 Metropolitan Medical Center, Manila, Philippines; 95 University of Puerto Rico, San Juan, Puerto Rico; 96 Research Center of Neurology, Moscow, Russia; 97 King Faisal Specialist Hospital and Research Center, Riyadh, Saudi Arabia; 98 King Abdullah International Medical Research Center, Jeddah, Saudi Arabia; 99 National Neuroscience Institute, Singapore, Singapore; 100 University of KwaZulu-Natal, Durban, South Africa; 101 University of Stellenbosch, Stellenbosch, South Africa; 102 Stellenbosch University, Stellenbosch, South Africa; 103 Seoul National University Hospital, Seoul, South Korea; 104 Yongin Severance Hospital, Seoul, South Korea; 105 Hospital Universitario de Burgos, Burgos, Spain; 106 University Hospital Mutua Terrassa, Barcelona, Spain; 107 Institut de Recerca Sant Joan de Déu, Barcelona, Spain; 108 Research Institute Germans Trias i Pujol, Badalona, Spain; 109 University Hospital Germans Trias i Pujol, Badalona, Spain; 110 Faculty of Medicine, University of Khartoum, Khartoum, Sudan; 111 Inselspital, Bern University Hospital, University of Bern, Bern, Switzerland; 112 National Taiwan University Hospital, Taipei City, Taiwan; 113 Chang Gung Memorial Hospital, Taoyuan City, Taiwan; 114 National Taiwan University, Taipei City, Taiwan; 115 Chang Gung University, Taoyuan City, Taiwan; 116 National Institute Mongi Ben Hamida of Neurology, Tunis, Tunisia; 117 Mongi Ben Hmida National Institute of Neurology, Tunis, Tunisia; 118 Koç University, Istanbul, Turkey; 119 Şişli Etfal Training and Research Hospital, Istanbul, Turkey; 120 University College London, London, UK; 121 University of Plymouth, Plymouth, UK; 122 Parkinson’s UK, London, UK; 123 University of Glasgow, Glasgow, UK; 124 Queen Mary University of London, London, UK; 125 Cardiff University, Cardiff, UK; 126 Royal Veterinary College, University of London, London, UK; 127 University of Bristol, Bristol, UK; 128 Cure Parkinson’s Trust, London, UK; 129 University of Cincinnati, Cincinnati, OH USA; 130 The Michael J. Fox Foundation for Parkinson’s Research, New York, NY USA; 131 Augusta University / University of Georgia Medical Partnership, Athens, GA USA; 132 Mid-Atlantic Permanente Medical Group, Rockville, MD USA; 133 Washington University in St. Louis, St. Louis, MO USA; 134 National Institutes of Health, Bethesda, MD USA; 135 Indiana University, Bloomington, IN USA; 136 Rush University Medical Center, Chicago, IL USA; 137 Kaiser Permanente, Oakland, CA USA; 138 Aligning Science Across Parkinson’s, Washington, DC USA; 139 Banner Sun Health Research Institute, Sun City, AZ USA; 140 Michigan State University, East Lansing, MI USA; 141 Northwestern University, Chicago, IL USA; 142 Baylor College of Medicine, Houston, TX USA; 143 Baylor College of Medicine/Texas Children’s Hospital, Houston, TX USA; 144 University of Miami Miller School of Medicine, Miami, FL USA; 145 Beth Israel Deaconess Medical Center, Boston, MA USA; 146 NorthShore University HealthSystem, Evanston, IL USA; 147 Institute for Neurodegenerative Disorders, New Haven, CT USA; 148 University of Pittsburgh, Pittsburgh, PA USA; 149 University of Alabama at Birmingham, Birmingham, AL USA; 150 University of Maryland School of Medicine, Baltimore, MD USA; 151 University of Michigan, Ann Arbor, MI USA; 152 Columbia University Irving Medical Center, New York, NY USA; 153 James J. Peters VA Medical Center, Bronx, NY USA; 154 University of Chicago, Chicago, IL USA; 155 Indiana University School of Medicine, Indianapolis, IN USA; 156 Hue University, Huế, Vietnam; 157 University of Zambia, Lusaka, Zambia

**Keywords:** Diseases, Genetics, Neurology, Neuroscience

Correction to: *npj Parkinson’s Disease* 10.1038/s41531-025-01180-z, published online 02 December 2025

In this article, the author list for the Global Parkinson’s Genetic Program (GP2) consortium was incomplete. The full list of GP2 consortium members has now been provided in the Supplementary Information.

