# Supplementary material for: TMEM175, SCARB2 and CTSB associations with Parkinson’s disease risk across populations
Source: NPJ Parkinsons Dis. 2025 Dec 2;11:348. doi: 10.1038/s41531-025-01180-z (PMC12678402; doi:10.1038/s41531-025-01180-z)
Supplement: Supplementary file 1 — supplementary material [file 41531_2025_1180_MOESM1_ESM.pdf]

Supplementary tables and figures

Supplementary Table 1a. Association analysis of *TMEM175* p. Met393Thr and p. Gln65Pro across all ten populations.

Ancestry	*TMEM175* p. Met393Thr (rs34311866, 4:958159: T:C)					*TMEM175* p. Gln65Pro (rs34884217, 4:950422: A:C)				
	MAF (cases)	MAF (controls)	P-value	FDR corrected P-value	OR (95%CI)	MAF (cases)	MAF (controls)	P-value	FDR corrected P-value	OR (95%CI)
AAC	0.051	0.051	0.18	0.41	0.71 (0.43-1.17)	0.027	0.024	0.75	0.84	0.90 (0.45-1.77)
AFR	NA	NA	NA	NA	NA	NA	NA	NA	NA	NA
AJ	0.35	0.28	2.51e-03*	0.023*	1.43 (1.13-1.80)	0.082	0.10	0.042*	0.13	0.70 (0.49-0.99)
AMR	0.088	0.040	0.020*	0.088	1.36 (1.05-1.75)	0.062	0.032	0.85	0.91	1.03 (0.77-1.36)
CAS	0.22	0.22	0.64	0.81	1.06 (0.82-1.37)	0.037	0.029	0.46	0.64	1.22 (0.72-2.05)
EAS	0.15	0.14	0.086	0.23	1.14 (0.98-1.34)	NA	NA	NA	NA	NA
EUR	0.21	0.18	6.07e-16*	2.79e-14*	1.25(1.19-1.32)	0.093	0.11	1.70e-04*	3.91e-03*	0.88(0.82-0.94)
MDE	0.23	0.24	0.019*	0.088	1.30 (1.04-1.63)	0.061	0.087	0.019*	0.088	0.66 (0.47-0.93)
SAS	0.38	0.28	8.88e-04*	0.010*	1.83(1.28-2.61)	0.024	0.019	0.41	0.64	1.75(0.46-6.57)
CAH	0.15	0.16	0.44	0.64	0.88(0.64-1.22)	0.070	0.050	0.37	0.64	1.24 (0.77-1.98)

Abbreviations: AAC, African American; AFR, African; AJ, Ashkenazi Jewish; AMR, Latino and indigenous Americas; CAS, Central Asian; EAS, East Asian; EUR, European; MDE, Middle Eastern; SAS, South Asian; CAH, Complex Admixture History. MAF, minor allele frequency. OR, odds ratio. CI, Confidence Interval. NA not assessed because MAF <0.01. * p value < 0.05.
Location of variants are based on GRCh38/hg38. P values were corrected for multiple testing across ten ancestries and five genetic variants using the Benjamini–Hochberg method to control the false discovery rate.

Supplementary Table 1b. Association analysis of *SCARB2* rs6812193 and rs6825004 across all ten populations

Ancestry	*SCARB2* rs6812193(4:76277833:C: T)					*SCARB2* rs6825004(4:76189212:C: G)				
	MAF (cases)	MAF (controls)	P-value	FDR corrected P-value	OR (95%CI)	MAF (cases)	MAF (controls)	P-value	FDR corrected P-value	OR (95%CI)
AAC	0.40	0.46	0.43	0.64	0.86(0.72-1.03)	0.23	0.21	0.96	0.96	1.01(0.78-1.30)
AFR	0.46	0.45	0.76	0.84	1.02(0.90-1.15)	0.18	0.19	0.45	0.64	1.06(0.91-1.25)
AJ	0.29	0.33	0.075	0.22	0.82(0.65-1.02)	0.40	0.39	0.77	0.84	1.03(0.84-1.27)
AMR	0.22	0.15	0.020*	0.088	0.83(0.71-0.97)	0.49	0.43	0.69	0.81	1.02(0.91-1.15)
CAS	0.21	0.21	0.69	0.81	1.05(0.83-1.32)	0.32	0.37	0.12	0.31	0.85(0.69-1.04)
EAS	0.076	0.078	0.27	0.52	0.90(0.74-1.09)	0.36	0.34	0.23	0.48	1.07(0.96-1.19)
EUR	0.35	0.36	7.81e-03*	0.060	0.94(0.90-0.98)	0.30	0.31	0.036*	0.13	0.95(0.91-1.00)
MDE	0.42	0.41	0.48	0.65	0.94(0.78-1.12)	0.37	0.39	0.023*	0.088	0.80(0.66-0.97)
SAS	0.27	0.29	0.39	0.64	0.85(0.60-1.22)	0.23	0.23	0.92	0.96	0.98(0.69-1.39)
CAH	0.31	0.35	0.15	0.36	0.84(0.67-1.07)	0.29	0.28	0.042*	0.13	0.78(0.62-0.99)

Abbreviations: AAC, African American; AFR, African; AJ, Ashkenazi Jewish; AMR, Latino and indigenous Americas; CAS, Central Asian; EAS, East Asian; EUR, European; MDE, Middle Eastern; SAS, South Asian; CAH, Complex Admixture History. MAF, minor allele frequency. OR, odds ratio. CI, Confidence Interval. NA, not available. * means p value < 0.05.
Location of variants are based on GRCh38/hg38. P values were corrected for multiple testing across ten ancestries and five genetic variants using the Benjamini–Hochberg method to control the false discovery rate.

Supplementary Table 1c. Association analysis of *CTSB* rs1293298 across all ten populations

Ancestry	MAF (cases)	MAF (controls)	P-value	FDR corrected P-value	OR (95%CI)
AAC	0.29	0.33	0.20	0.44	0.86(0.68-1.08)
AFR	0.33	0.31	0.26	0.52	1.08(0.95-1.23)
AJ	0.29	0.31	0.29	0.53	0.88(0.71-1.11)
AMR	0.14	0.081	0.69	0.81	0.96(0.79-1.16)
CAS	0.11	0.13	0.46	0.64	0.89(0.66-1.21)
EAS	NA	NA	NA	NA	NA
EUR	0.24	0.25	5.82e-04*	8.92e-03*	0.92(0.87-0.96)
MDE	0.27	0.28	0.53	0.70	0.94(0.76-1.15)
SAS	0.17	0.17	0.94	0.96	1.02(0.67-1.55)
CAH	0.21	0.26	0.021*	0.088	0.73(0.56-0.95)

Abbreviations: AAC, African American; AFR, African; AJ, Ashkenazi Jewish; AMR, Latino and indigenous Americas; CAS, Central Asian; EAS, East Asian; EUR, European; MDE, Middle Eastern; SAS, South Asian; CAH, Complex Admixture History. MAF, minor allele frequency. OR, odds ratio. CI, Confidence Interval. NA, not available. * means p value < 0.05.
Location of variants are based on GRCh38/hg38. P values were corrected for multiple testing across ten ancestries and five genetic variants using the Benjamini–Hochberg method to control the false discovery rate.

Supplementary Table 2. eQTL analysis of *SCARB2* rs11547135 in public datasets.

Variant	GP2-EUR				eQTLGen in blood						GTEx in brain tissue		
									PP.H4				
	Beta	SE	P-value	N	Beta	SE	P-value	N	p12 = 1e-05	p12 = 5e-06	Beta	P-value	N
SCARB2 rs11547135	0.10	0.023	1.01e-05*	33,870	0.087	0.0056	1.38e-53*	31,477	99.42%	98.85%	0.37	3.32e-06*	183

Abbreviations: Beta, effect size; SE, standard error; N, sample size; PP.H4, posterior probability for H4, which shows probability of a shared causal variant. p12, prior probability that a SNP is associated with both traits.

Supplementary Table 3a. SKAT-O analysis of *TMEM175* with p. Met393Thr and p. Gln65Pro included as covariates.

Ancestry	Variants	*TMEM175* Num Var	rho	P-value	FDR P-value corrected
AAC	exonic	59	0	0.42	0.73
	Nonsyn and LoF	38	0	0.62	0.74
AFR	exonic	76	1	0.87	0.87
	Nonsyn and LoF	47	1	0.70	0.74
AJ	exonic	24	0	0.21	0.57
	Nonsyn and LoF	15	0	0.24	0.57
AMR	exonic	72	0	0.53	0.74
	Nonsyn and LoF	42	1	0.14	0.53
CAS	exonic	39	1	0.69	0.74
	Nonsyn and LoF	26	NA	NA	NA
EAS	exonic	52	1	0.26	0.57
	Nonsyn and LoF	34	1	0.044*	0.39
EUR	exonic	128	0	0.062	0.39
	Nonsyn and LoF	69	0	0.14	0.53
MDE	exonic	47	0	0.36	0.68
	Nonsyn and LoF	24	0	0.61	0.74
SAS	exonic	25	0	0.47	0.74
	Nonsyn and LoF	18	1	0.61	0.74
CAH	exonic	47	1	0.27	0.57
	Nonsyn and LoF	30	1	5.98e-03*	0.11

Abbreviations: AAC, African American; AFR, African; AJ, Ashkenazi Jewish; AMR, Latino and indigenous Americas; CAS, Central Asian; EAS, East Asian; EUR, European; MDE, Middle Eastern; SAS, South Asian; CAH, Complex Admixture History; Nonsyn, non-synonymous variants; LoF, and Loss of function; NA, not available. NumVar, number of variants. P values were corrected for multiple testing across ten ancestries and two variant categories using the Benjamini–Hochberg method to control the false discovery rate.

Supplementary Table 3b. SKAT test of *SCARB2* with GP2 dataset based on significant results from Skat-O analysis.

Ancestry	Variants	Test	NumVar	P-value
AAC	exonic	SKAT	21	8.90e-04*
AJ	exonic	SKAT	5	0.021*
EAS	exonic	SKAT	17	0.013*
	Nonsyn and LoF	SKAT	12	0.011*

Abbreviations: AAC, African American; AJ, Ashkenazi Jewish; EAS, East Asian; Nonsyn, non-synonymous variants; LoF, and Loss of function; NumVar, number of variants.

Supplementary Table 4. Numbers of *GBA1* carriers (removed) and non-*GBA1* individuals in the GP2 Neurobooster cohort across ancestries.

N	EUR	AAC	AFR	AJ	AMR	CAS	EAS	MDE	SAS	CAH
GBA1 carriers	2,137	31	78	230	65	31	7	12	4	35
non-*GBA1*	33,870	1,125	3,316	1,693	3,319	1,312	4,975	1,275	570	981

Abbreviations: AAC, African American; AFR, African; AJ, Ashkenazi Jewish; AMR, Latino and indigenous Americas; CAS, Central Asian; EAS, East Asian; EUR, European; MDE, Middle Eastern; SAS, South Asian; CAH, Complex Admixture History; N indicates sample size, and only individuals with available covariates were included.

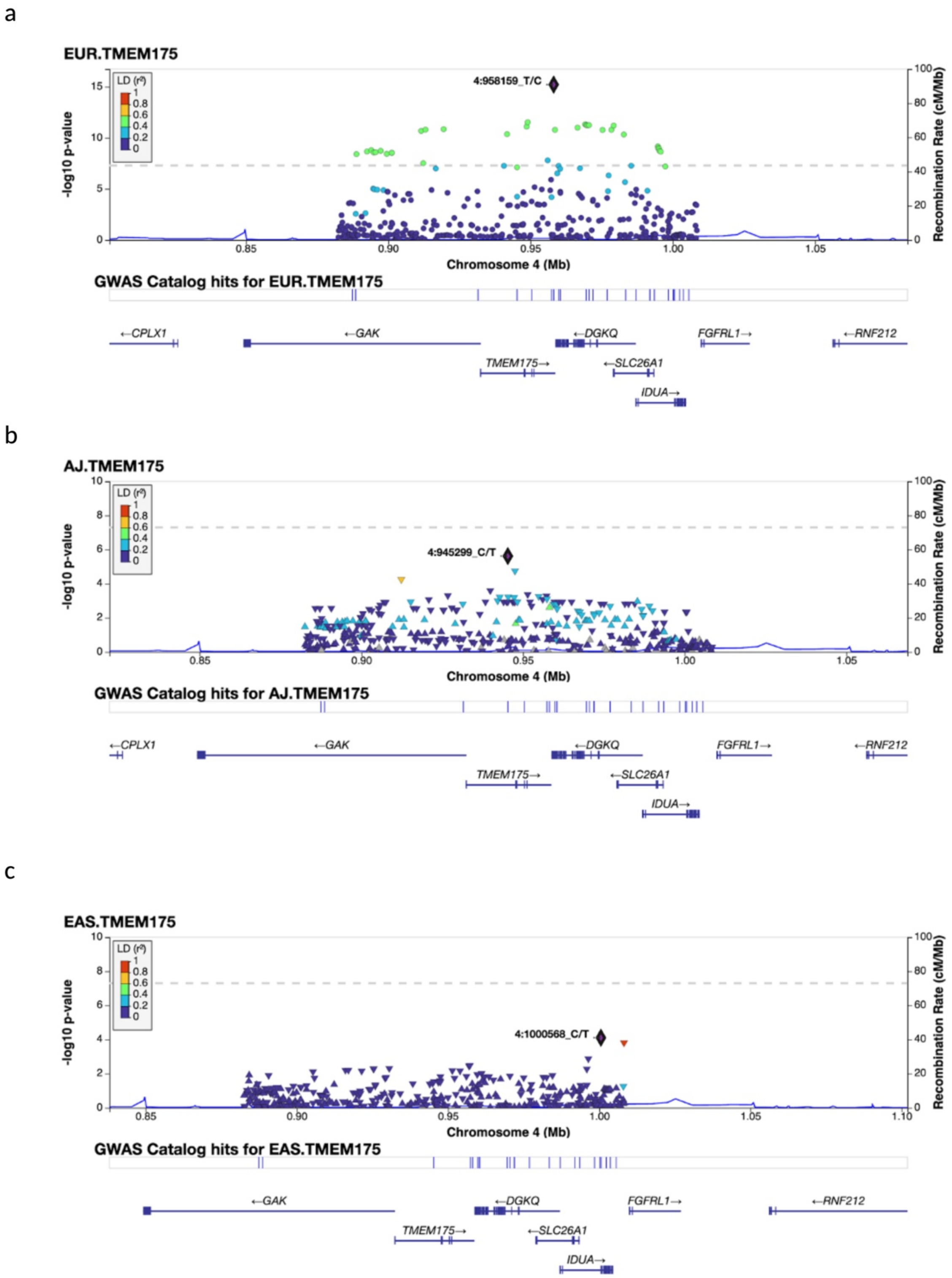
a
EUR.TMEM175
4:958159_T/C
LD (r²)
1
0.8
0.6
0.4
0.2
0
-log10 p-value
Recombination Rate (cM/Mb)
Chromosome 4 (Mb)
GWAS Catalog hits for EUR.TMEM175
←CPLX1
←GAK
←DGKQ
FGFRL1→
←RNF212
TMEM175→
←SLC26A1
IDUA→
b
AJ.TMEM175
4:945299_C/T
LD (r²)
-log10 p-value
Recombination Rate (cM/Mb)
Chromosome 4 (Mb)
GWAS Catalog hits for AJ.TMEM175
←CPLX1
←GAK
←DGKQ
FGFRL1→
←RNF212
TMEM175→
←SLC26A1
IDUA→
c
EAS.TMEM175
4:1000568_C/T
LD (r²)
-log10 p-value
Recombination Rate (cM/Mb)
Chromosome 4 (Mb)
GWAS Catalog hits for EAS.TMEM175
←GAK
←DGKQ
FGFRL1→
←RNF212
TMEM175→
←SLC26A1
IDUA→

Supplementary Fig. 1: Association signals at the *TMEM175* locus across distinct GP2 cohorts. a) GP2-EUR cohort: p. Met393Thr (4:958159); b) GP2-AJ cohort: rs6599388 (4:945299); c) GP2-EAS cohort: rs3755956 (4:1000568).

a

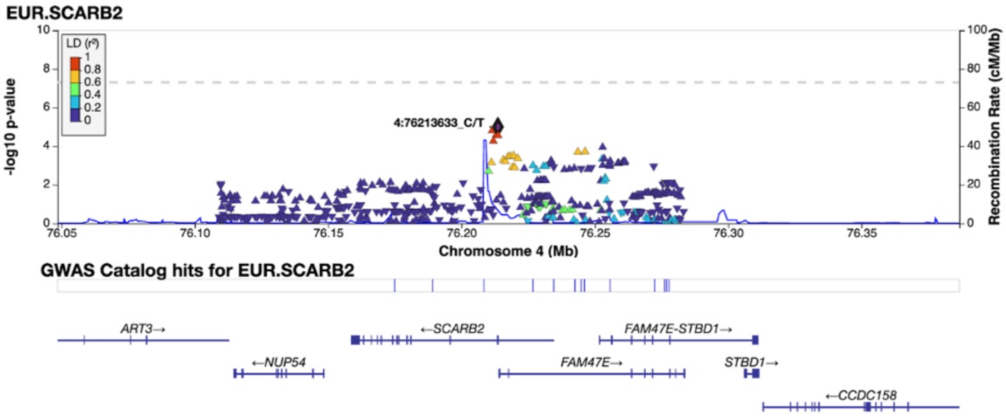
EUR.SCARB2
LD (r²)
-log10 p-value
Recombination Rate (cM/Mb)
4:76213633_C/T
Chromosome 4 (Mb)
GWAS Catalog hits for EUR.SCARB2
ART3→
←NUP54
←SCARB2
FAM47E-STBD1→
FAM47E→
STBD1→
←CCDC158

b

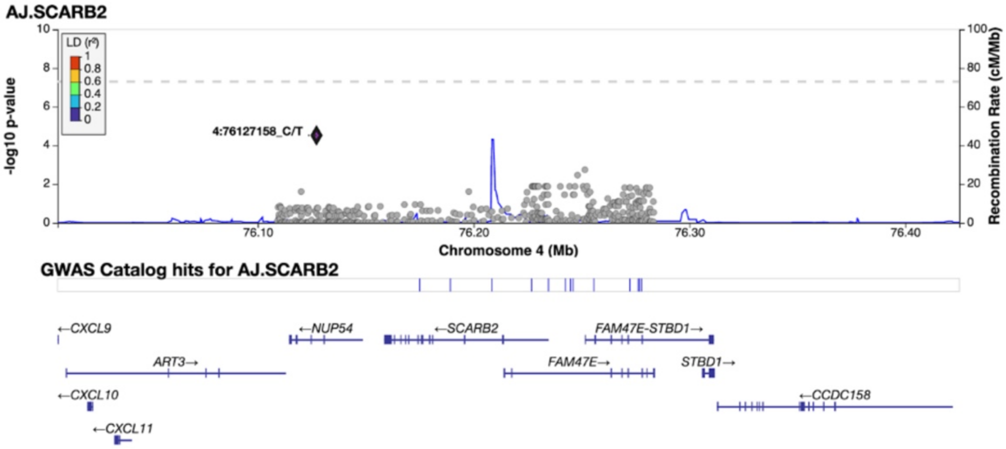
AJ.SCARB2
LD (r²)
-log10 p-value
Recombination Rate (cM/Mb)
4:76127158_C/T
Chromosome 4 (Mb)
GWAS Catalog hits for AJ.SCARB2
←CXCL9
←NUP54
←SCARB2
FAM47E-STBD1→
ART3→
FAM47E→
STBD1→
←CXCL10
←CCDC158
←CXCL11

c

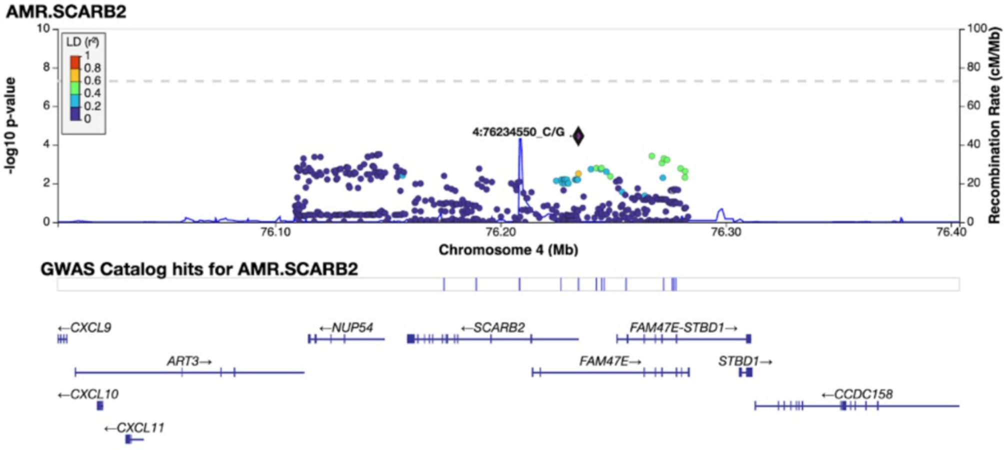
AMR.SCARB2
LD (r²)
-log10 p-value
Recombination Rate (cM/Mb)
4:76234550_C/G
Chromosome 4 (Mb)
GWAS Catalog hits for AMR.SCARB2
←CXCL9
←NUP54
←SCARB2
FAM47E-STBD1→
ART3→
FAM47E→
STBD1→
←CXCL10
←CCDC158
←CXCL11

Supplementary Fig. 2: Association signals at the *SCARB2* locus across distinct GP2 cohorts. a) GP2-EUR cohort: rs11547135(4:76213633); b) GP2-AJ cohort: rs530111925(4:76127158); c) GP2-AMR cohort: rs73828719(4:76234550).

a

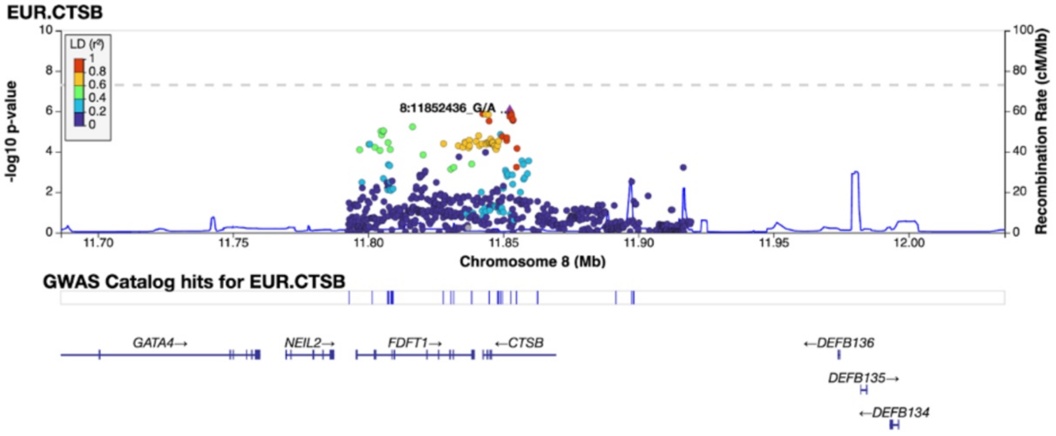

b

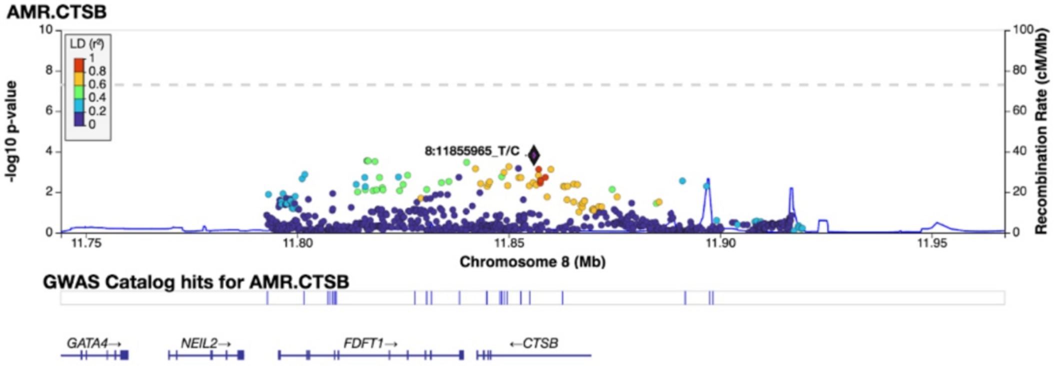

Supplementary Fig. 3: Association signals at the *CTSB* locus across distinct GP2 cohorts. a) GP2-EUR cohort: rs1293289(8:11852436); b) GP2-AMR cohort: rs73551266 (8:11855965).

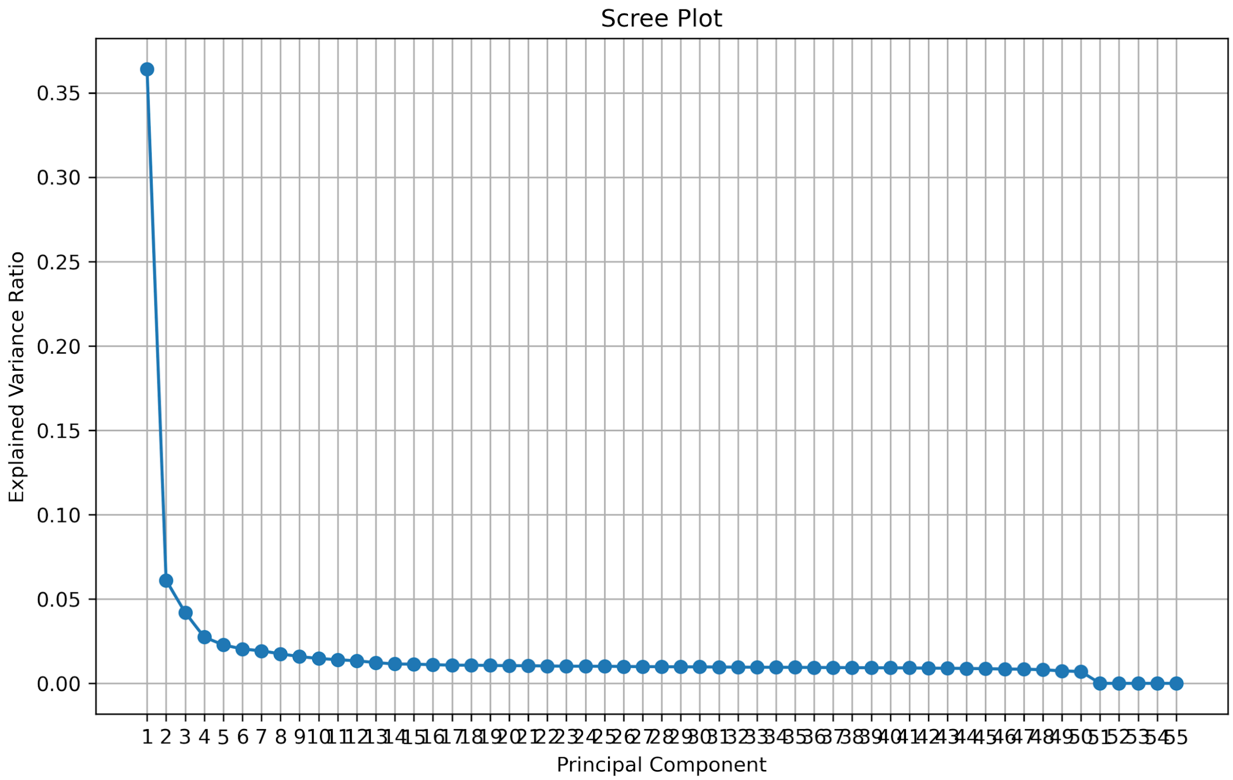

Supplementary Fig. 4: Scree plot of principle components in European population.

STROBE Statement—Checklist of items that should be included in reports of ***case-control studies***

	Item No	Recommendation
**Title and abstract**	1	(a) Indicate the study's design with a commonly used term in the title or the abstract<br>**Abstract line 2-4.**
		(b) Provide in the abstract an informative and balanced summary of what was done and what was found<br>**Abstract, full paragraph.**
**Introduction**		
Background/rationale	2	Explain the scientific background and rationale for the investigation being reported<br>**Introduction, paragraph 1-4**
Objectives	3	State specific objectives, including any prespecified hypotheses<br>**Introduction, paragraph 5**
**Methods**		
Study design	4	Present key elements of study design early in the paper<br>**Methods: subjects**
Setting	5	Describe the setting, locations, and relevant dates, including periods of recruitment, exposure, follow-up, and data collection<br>**Methods: subjects**
Participants	6	(a) Give the eligibility criteria, and the sources and methods of case ascertainment and control selection. Give the rationale for the choice of cases and controls<br>**Methods: subjects**
		(b) For matched studies, give matching criteria and the number of controls per case<br>**Methods: subjects**
Variables	7	Clearly define all outcomes, exposures, predictors, potential confounders, and effect modifiers. Give diagnostic criteria, if applicable<br>**Methods: statistical analysis**
Data sources/ measurement	8*	For each variable of interest, give sources of data and details of methods of assessment (measurement). Describe comparability of assessment methods if there is more than one group<br>**Methods: subjects and statistical analysis**
Bias	9	Describe any efforts to address potential sources of bias<br>**Methods: statistical analysis**
Study size	10	Explain how the study size was arrived at<br>**Methods: subjects**
Quantitative variables	11	Explain how quantitative variables were handled in the analyses. If applicable, describe which groupings were chosen and why<br>**Methods: statistical analysis**
Statistical methods	12	(a) Describe all statistical methods, including those used to control for confounding<br>**Methods: statistical analysis**
		(b) Describe any methods used to examine subgroups and interactions<br>**Not applicable**
		(c) Explain how missing data were addressed<br>**Methods: subjects**
		(d) If applicable, explain how matching of cases and controls was addressed
		(e) Describe any sensitivity analyses<br>**Methods: statistical analysis**

Results		
Participants	13*	(a) Report numbers of individuals at each stage of study—eg numbers potentially eligible, examined for eligibility, confirmed eligible, included in the study, completing follow-up, and analysed **Figure 1, Results and Tables**
		(b) Give reasons for non-participation at each stage **Methods: subjects**
		(c) Consider use of a flow diagram **Figure 1**
Descriptive data	14*	(a) Give characteristics of study participants (eg demographic, clinical, social) and information on exposures and potential confounders **Methods: statistical analysis**
		(b) Indicate number of participants with missing data for each variable of interest **Methods: subjects**
Outcome data	15*	Report numbers in each exposure category, or summary measures of exposure **Figure 1, Methods: subjects**
Main results	16	(a) Give unadjusted estimates and, if applicable, confounder-adjusted estimates and their precision (eg, 95% confidence interval). Make clear which confounders were adjusted for and why they were included **Figures and Tables, Methods and Results**
		(b) Report category boundaries when continuous variables were categorized **Not applicable**
		(c) If relevant, consider translating estimates of relative risk into absolute risk for a meaningful time period **Not applicable**

Other analyses	17	Report other analyses done—eg analyses of subgroups and interactions, and sensitivity analyses<br>**Methods: statistical analysis, Figures and Tables**
**Discussion**		
Key results	18	Summarise key results with reference to study objectives<br>**Discussion: paragraph 1**
Limitations	19	Discuss limitations of the study, taking into account sources of potential bias or imprecision. Discuss both direction and magnitude of any potential bias<br>**Discussion: paragraph 10**
Interpretation	20	Give a cautious overall interpretation of results considering objectives, limitations, multiplicity of analyses, results from similar studies, and other relevant evidence<br>**Discussion: paragraph 2-9**
Generalisability	21	Discuss the generalisability (external validity) of the study results<br>**Discussion: paragraph 11**
**Other information**		
Funding	22	Give the source of funding and the role of the funders for the present study and, if applicable, for the original study on which the present article is based<br>**Acknowledgements**

*Give information separately for cases and controls.

Note: An Explanation and Elaboration article discusses each checklist item and gives methodological background and published examples of transparent reporting. The STROBE checklist is best used in conjunction with this article (freely available on the Web sites of PLoS Medicine at http://www.plosmedicine.org/, Annals of Internal Medicine at http://www.annals.org/, and Epidemiology at http://www.epidem.com/). Information on the STROBE Initiative is available at http://www.strobe-statement.org.

Author information

Authors and Affiliations

Wenhua Sun[1], Claudia Schulte[1], Thomas Gasser[1*], Manuela Tan[2]

Consortia

the Global Parkinson's Genetics Program (GP2)

Kathrin Brockmann[1], Isabel Wurster[1], Lasse Pihlstrøm[2], Ingeborg Haugesag Lie[2], Solveig E J Dalbro[2], Ellen Hoven Maurtveten[2], Yasser Mecheri[3], Bouchetara Mohamed Sofiane[4], Benhassine Traki[5], Emilia M Gatto[6], Marcelo Kauffman[7], Federico Capparelli[8], Maria Valentina Muller [9], Marcela Susana Tela[10], Adamec, Dario Sergio [11], Cesar Luis Avila[12], Samson Khachatryan [13], Zaruhi Tavadyan [13], Mariam Isayan [13], Claire E Shepherd [14], Simon Rowe[14], Dennis Yeow[14], Carolyn Sue[14], Kishore Kumar[15], Melina Ellis [16], Miguel E. Rentería [17], Sulev Koks[18], Victor Flores Ocampo[19], Nicholas G. Martin[19], Luis M. García-Marín[19], Christine Wools[20], Keren Aliza Weiss[21], Amanda Willis[21], Steven He[21], Sue-Faye Siow[22], Ryan L Davis[23], Robert Arthur Wilcox[24], Denise Howting[25], Jack David Price[26], Pak Leng Cheong[27], Michel Tchan[28], Mary-Anne Young[29], Catriona Mclean[30], Hugo Morales Briceño[31], Thomas Kimber[32], Kathy H. C. Wu[33], John O'Sullivan[34], Lewis M Singleton[35], Laura Ivete Rudaks[36], Alexander Zimprich[37], Kanan Jafarov[38], Kenan Ceferov[39], Imran Sarker[40], David Crosiers[41], Artur F. Schumacher-Schuh[42], Carlos Rieder[43], Paula Saffie Awad[44], Vitor Tumas[45], Sarah Camargos[46], Lucas Faria Costa[46], Pedro Braga Neto[47], Oury Monchi[48], Edward Fon[49], Ziv Gan-Or[49], Meron Teferra[49], Konstantin Senkevich[49], Robert Thibault[50], Anthony Lang[51], Marcelo Miranda[52], Ana Belen Miranda Cortes[52], Maria Leonor Bustamante[53], Juan Cristobal Nuñez[54], Boris Lucero[55], Alicia Colombo[56], Maria Teresa Muñoz Personal[57], Benjamín Pizarro-Galleguillos[57], Eduardo Pérez Palma[58], Pedro Chana-Cuevas[59], María Eugenia Contreras Pinto[60], Francisca Canals[61], Patricio Alejandro Olguín Aguilera[62], Elias Fernandez-Toledo[63], Benjamín Pizarro Galleguillos[64], Beisha Tang[65], Huifang Shang[66], Jifeng Guo[67], Piu Chan[68], Wei Luo[69], Zhenhua Liu[70], Germaine Hiu-Fai Chan[71], Nelson Yuk-Fai Cheung[71], Nancy Ip[72], Phillip Chan[72], Xiaopu Zhou[72], Gonzalo Arboleda[73], Tatiana Lopez-Gonzalez[73], Jorge Orozco[74], Beatriz Munoz Ospina[74], David Antonio Pineda-Salazar[75], Carlos Velez-Pardo[76], Marlene Jimenez-Del-Rio[76], Sonia Moreno Masmela[76], Alvaro Hernandez[77], Per Borghammer[78], Mohamed Salama[79], Walaa A. Kamel[80], Tatiana Ascencio[81], Oscar Peña-Rodas[82], Susana Lissette Peña Martínez[83], Yared Z. Zewde[84], Alexis Brice[85], Yves Agid[85], Alexandra Durr[85], Aymeric Lanore[85], Jean-Christophe Corvol[86], Mari Vidailhet[87], Mathieu Anheim[88], Louise-Laure Mariani[89], Rascol[90], Ory Magne Fabienne[91], Suzanne Lesage[92], Defebvre Luc[93], Tesson Christelle[94], Philippe Damier[95], François Tison[96], Stéphane Thobois[97], Jean-Luc Houeto[98], Brefel Corbon Christine[99], Sara Sambin[100], Mariam Kekenadze[101], Maia Beridze[101], Irine Khatiasvili[102], Sophia Sopromadze[103], Irine Khatiasvilli[103], Mariam Mshvenieradze[103], Alexander Tsiskaridze[103], Marika Megrelishvili[104], Ana Westenberger[105], Carolin Gabbert[105], Alexander Balck[105], Christine Klein[105], Eva-Juliane Vollstedt[105], Harutyun Madoev[105], Joanne Trinh[105], Katja Lohmann[105], Inke

König[105], Teresa Kleinz[105], Norbert Brüggemann[105], Theresa Lüth[105], Anastasia Illarionova[106], Brit Mollenhauer[107], Franziska Hopfner[108], Günter Höglinger[108], Manu Sharma[109], Sergiu Groppa[110], Zih-Hua Fang[111], Karl Heilbron[112], Daniela Berg[113], Bernhard Haslinger[114], Konstantin Kufer[115], Antonia Maria Buchal[116], Matthias Höllerhage[117], Florian Wegner[117], Martin Klietz[117], Nils Schroeter[118], Christian Beetz[119], Krishnakumar Kandaswamy[120], Eva Schäffer[121], Kirsten Zeuner[121], Gregor Kuhlenbäumer[121], Peter Bauer[122], Albert Akpalu[123], Momodou Cham[124], Vida Obese[125], Georgia Xiroemisiou[126], Georgios Hadjigeorgiou[126], Efthymios Dadiotis[126], Ioannis Dagklis[127], Ioannis Tarmanas[128], Leonidas Stefanis[129], Maria Stamelou[130], Tsamis Konstantinos[131], Konitsiotis Spyridon[131], Foivos S. Kanellos[131], Iro Boura[132], Cleanthe Spanaki[132], Maria[133], Lina Florentin[133], Maria Makrygianni[133], Alex Medina[134], Evelin Álvarez Herrera[135], Heike Hesse Joya[136], Reyna M. Durón[137], Eduardo Jose Ponce Murillo[137], Glenda Oliva Fuentes[138], Kari Stefansson[139], Hreinn Stefansson[139], Vala Palmadottir[139], Astros Th. Skuladottir[139], Asha Kishore[140], Divya Kp[141], Pramod Pal[142], Prashanth Lingappa Kukkle[143], Roopa Rajan[144], Rupam Borgohain[145], Mehri Salari[146], Tamara Shiner[147], Avner Thaler[147], Noa Bregman[148], Andrea Quattrone[149], Enza Maria Valente[150], Micol Avenali[150], Michele Terzaghi[150], Grazia Annesi[151], Lucilla Parnetti[152], Monica Gagliardi[153], Jolanda Buonocore[153], Radha Procopio[153], Tommaso Schirinzi[154], Caterina Galandra[155], Ilaria Palmieri[155], Anna De Rosa[156], Rosangela Ferese[157], Manabu Funayama[158], Nobutaka Hattori[159], Tomotaka Shiraishi[160], Kensuke Daida[161], Altynay Karimova[162], Gulnaz Kaishibayeva[162], Guzel Shiderova[162], Aigerim Utegenova[163], Aigul. P. Yermagambetova[163], Alima A. Khamidulla[163], Zhanylsyn U.Urasheva[163], Gulnar B. Kabdrakhmanova[163], Vadim Akmetzhanov[164], Seitzhan Aidarov[165], Tautanova Raushan[166], Dinara Alzhanova[166], Bagzhan Syzdykova[166], Zhanybek Myrzayev[167], Saltanat Abdraimova[168], Nazira Zharkinbekova[168], Chingiz Shashkin[169], Talgat Khaibullin[170], Altynay Talgatkyzy[170], Cholpon Shambetova[171], Rejko Krüger[172], Patrick May[172], Ai Huey Tan[173], Azlina Ahmad-Annuar[173], Shen-Yang Lim[173], Yi Wen Tay[173], Lim Kai-Shi[173], Azalea Tenerife Pajo[173], Tzi Shin Toh[173], Mohamed Ibrahim Norlinah[174], Nor Azian Abdul Murad[175], Shahrul Azmin[176], Wael Mohamed[177], Chia Yuen Kang[178], Joshua Ooi Chin Ern[178], Khairul Azmi Ibrahim[179], Ahmad Shahir Bin Mawardi[180], Lim Thien Thien[181], Daniel Martinez-Ramirez[182], Paula Reyes-Pérez[183], Alejandra Medina Rivera[183], Edith Janeth Gaspar Martínez[183], Nancy Monroy Jaramillo[184], Mayela Rodríguez-Violante[184], Amin Cervantes-Arriaga[184], Nadia Alejandra Gandarilla Martinez[185], Ingrid Estrada-Bellmann[186], Araliz Puente[187], Ana Paula Angulo Arrieta[188], Eugenia Morelos Figaredo[189], Karla Salinas Barboza[190], Dante Bernardo Oropeza Canto[191], Ana Jimena Hernández-Medrano[192], Alejandra E. Ruiz-Contreras[193], Alejandra Lázaro-Figueroa[194], Bayasgalan Tserensodnom[195], Khosbayar Tulgaa[195], Oyujin Ulziibaatar[195], Ahmed Bouhouche[196], Mossafa Hossain[197], Rajeev Ojha[198], Wilma Van De Berg[199], Bas Bleom[200], Bart Van De Warrenburg[201], Lisette Charbonnier[202], Tim J. Anderson[203], Toni L. Pitcher[203], Daniel Jeremy Myall[204], John C. Dalrymple-Alford[205], Arinola Sanyaolu[206], Nijideka Okubadejo[206], Lara Ojo[206], Francis Ojini[206], Oluwadamilola Ojo[207], Simon Izuchukwu Ozomma[208], Kolawole Wahab[209], Wemimo Alaofin[209], Oladunni Abiodun[210], Olanike Odeniyi[210], Sani Abubakar[211], Fatimah Abdulali[212], Nkechi Obianozie[212], Charles Achoru[213], Godwin Osaigbovo[213], Osigwe Agabi[214], Roosevelt Anyanwu[214], Ismaila Ishola[214], Francscisca Nwaokorie[214], Uchechi Agulanna[215], Daniel Ezuduemoih[215], Erica Ikwenu[215], Rufus Akinyemi[216], Ifeyinwa Ani-Osheku[217], Ohwotemu Arigbodi[218], Ewere Marie Ogbimi[218], Abiodun Bello[219], Cyril Erameh[220], Temitope Farombi[221], Fumilola Taiwo[221], Abdullahi Ibrahim[222], Ahmed Idowu[223], Frank Imarhiagbe[224], Francis Odiase[224], Emmanuel Iwuozo[225], Morenikeji Komolafe[226], Alero Nnama[227], Paul Nwani[228], Ernest

Nwazor[229], Yakubu Nyandaiti[230], Sarah Samuel[230], Yahaya Odiabo[231], Adebimpe Ogunmode[232], Raymond Owolabi[232], Rashidat Olanigan[233], Adedunni Olusanya[234], Chiamaka Okereke[235], Gerald Onwuegbuzie[236], Nosakhare Osemwegie[237], Olajumoke Oshinake[238], Folajimi Otubogun[239], Lukman Owolabi[240], Shyngle Oyakhire[241], Yusuf Zubair[241], Fadimatu Sa'Ad[242], Jodi Maple-Grødem[243], Shoaib Ur-Rehman[244], Mohamed Nour[245], Mario Cornejo-Olivas[246], Maria Leila Doquenia[247], Raymond Rosales[247], Gerard Saranza[248], Agata Gajos[249], Elena Iakovenko[250], Anna Gareeva[251], Gulnara Akhmadeeva[252], Irina Gilyazova[253], Bashayer Al Mubarak[254], Muhammad Umair[255], Nataša Dragašević Mišković[256], Andona Milovanović[256], Eng-King Tan[257], Jia Nee Foo[258], Elaine Chew[258], Vesna Van Midden[259], Ferzana Amod[260], Jonathan Carr[261], Soraya Bardien[262], Nikita Pillay[263], Kathryn Step[264], Riaan Van Coller[265], Beomseok Jeon[266], Yun Joong Kim[267], Jung Hwan Shin[268], Joowon Jang[268], Jee-Young Lee[269], Esther Cubo[270], Ignacio Alvarez[271], Janet Hoenicka[272], Katrin Beyer[273], Maria Teresa Periñan[274], Pilar Gómez Garre[274], Pablo Mir[274], Manuela San Eufrasio Martínez[274], Laura Muñoz Delgado[274], Rafael Díaz Belloso[274], Sergio García Díaz[274], Marta Bonilla Toribio[274], Dolores Buiza Rueda[274], Antonio Cristobal Luque Ambrosiani[274], Silvia Jesus Maestre[274], Daniel Macías García[274], Elena Ojeda Lepe[274], Rocío Pineda Sánchez[274], Ana Castellano Guerrero[274], Astrid Daniela Adarmes Gómez[274], Cristina Pérez Calvo[274], Alejandro Salguero Oviedo[274], Lorena Clavijo Jiménez[274],Pau Pastor[275], Ruben Fernandez-Santiago[276], Celia Painous Marti[276], Almudena Sánchez-Gómez[276], Esteban Muñoz[276], Mario Ezquerra[277], Lola J. Díaz-Feliz[278], José Matías Arbelo González[279], Juan Carlos Martínez Castrillo[280], Marina Mata[281], Oriol De Fabregues[282], Lydia Vela-Desojo[283], Manuel Menendez Gonzalez[284], Yaroslau Compta[285], Alicia Garrido[286], Maria J Marti[287], Alexia T. Sánchez Reyes[288], Laia Muñoz Llahuna[289], Joaquim Amatell Escabies[289], Javier Pagonabarraga Mora[289], Ignacio Illán Gala[289], Sarah El-Sadig[290], Kajsa Brolin[291], Maria Swanberg[291], Per Svenningsson[292], Christiane Zweier[293], Paul Krack[293], Gerd Tinkhauser[294], Deborah Bartholdi[294], Chin-Hsien Lin[295], Ruey-Meei Wu[295], Hsiu-Chuan Wu[296], Yihru Wu[296], Pin-Jui Kung[297], Pin-Shiuan, Chen[298], Ganieva Manizha[299], Maksudjon Isrofilov[299], Rim Amouri[300], Samia Ben Sassi[300], Nabil Fatnassi Fatma[300], Amine Rachdi[300], Zakaria Saied[300], Rania Zouari[300], Chokri Mhiri[301], Mouna Ben Djebara[302], A. Nazlı Basak[303], Özgür Öztop Çakmak[303], Sibel Ertan[303], Rezzak Yilmaz[304], Binnur Çelik[304], Gençer Genç[305], Muhittin Cenk Akbostanci[306], Basar Bilgic[307], Bedia Samanci[307], Murat Emre[308], Haşmet Hanağasi[309], Aysegul Gunduz[310], Alastair Noyce[311], Sumit Dey[311], Spencer Finch[311], Alexandra Zirra[311], Ashvin Kuri[311], Sheena Waters[311], Laura Smith[311], Eduardo De Pablo-Fernández[311], Anisa Shahid[311], Cristina Simonet[311], Brook Huxford[311], Harneek Chohan[311], Sophie I Meyer[311], Charlotte Andrews[311], Emily Navarro Jones[311], Anette Schrag[312], Anthony Schapira[312], Eleanor J. Stafford[312], Henry Houlden[312], Huw R Morris[312], John Hardy[312], Kin Ying Mok[312], Mie Rizig[312], Nicholas Wood[312], Olaitan Okunoye[312], Rauan Kaiyrzhanov[312], Rimona Weil[312], Simona Jasaityte[312], Mina Ryten[312], Kailash Bhatia[312], Thomas Warner[312], Raquel Real[312], Matilda Lily Fenn[312], Lesley Yu Wu[312], Tessa Du Toit[312], Charlotte Dore[312], Oiher Serrano-Asensio[312], Marco Toffoli[312], Solomiia Bandrivska[312], Saiesha Dindayal[312], Camille Carroll[313], Donald Grosset[314], Nigel Williams[315], Valentina Escott-Price[315],Seth Love[316], Hamin Lee[317], Roger Barker[318], Caroline Williams-Gray[318], Michele Hu[319], Laura Parkkinen[319], Richard Walker[320], Steve Gentleman[321], Christian Lambert[321], Yen Tai[321], David Burn[322], Christopher M Morris[322], Deborah Attuah[323], Andrew C Robinson[324], Federico Roncaroli[324], Joshua Luc Isherwood Frost[325], Riona Fumi[326], Laura Pérez-Carbonell[327], Lara M. Lange[328], Alejandro Martínez-Carrasco[329], Angel Vinuela[330], Alyssa O'Grady[331], Bernadette Siddiqi[331], Bradford Casey[331], Brian Fiske[331], J Solle[331], Kaileigh Murphy[331], Maggie

Kuhl[331], Naomi Louie[331], Sohini Chowdhury[331], Todd Sherer[331], Ryan Pfingst[331], Debi Brooks[331], Zach Chaney[331], Conor Hennessey[331], Cassandra Barrett[331], Andrew B Singleton[332], Laurel Screven[332], Andrew K. Sobering[333], Cabell Jonas[334], Carlos Cruchaga[335], Laura Ibanez[335], Caroline B. Pantazis[336], Cornelis Blauwendraat[336], Claire Wegel[337], Dan Vitale[338], Faraz Faghri[338], Hampton Leonard[338], Mary B Makarious[338], Mathew Koretsky[338], Mike A. Nalls[338], Shannon Ballard[338], Deborah Hall[339], Dena Hernandez[340], Sara Bandres-Ciga[340], Spencer Grant[340], Ekemini Riley[341], Sonya Dumanis[341], Geidy E. Serrano[342], Thomas Beach[342], Ileana Lorenzini[342], Hirotaka Iwaki[343], Kristin S. Levine[343], Honglei Chen[344], Ignacio F. Mata[345], James B Leverenz[345], Thiago Peixoto Leal[345], Felipe Duarte-Zambrano[345], Emily Waldo[345], Ignacio Juan Keller Sarmiento[346], Niccolò Emanuele Mencacci[346], Jared Williamson[347], Jonggeol Jeff Kim[348], Joseph Jankovic[349], Chad Shaw[349], Joshua Shulman[350], Kamalini Ghosh Galvelis[351], Karen Nuytemans[352], Karl Kieburtz[353], Katerina Markopoulou[354], Kenneth Marek[355], Lana M. Chahine[356], Lauren Ruffrage[357], Marissa Dean[357], Lisa Shulman[358], Luca Marsili[359], Alberto J. Espay[359], Emily Hill[359], Megan J. Puckelwartz[360], Steven Lubbe[360], Roger Albin[361], Roy Alcalay[362], Ruth Walker[363], Tao Xie[364], Mahesh Padmanaban[364], Tatiana Foroud[365], Dana Lewis[366], Shreya Menon[367], Melissa Nirenberg[368], Rachel Saunders-Pullman[368], Sidra Aslam[369], Devin Sharp[370], Michiko Kimura Bruno[371], Matt Farrer[372], Ashley Rawls[372], Haydeh Payami[373], Elizabeth Disbrow[374], Randy Schekman[375], Un Kang[376], Zbigniew K. Wszolek[377], Cyrus Zabetian[378], Christine Swanson-Fischer[379], Beate Ritz[380], Bradley Boeve[381], Holly A. Shill[382], Erika Driver-Dunckley[383], Bruce A. Chase[384], Owen A. Ross[385], Michael Rose[386], Ariane Park[386], Victoria Klee[387], James C. Beck[388], Suzanne Judd[389], Daniel Weintraub[390], Vikas Kotagal[391], Nicolaas I. Bohnen[391], Prabesh Kanel[391], Chatkaew Pongmala[391], Erin Williams[392], Michael Henderson[392], Daniel C. Rohrer[392], Christina Missler[392], Alyssa Johansson[392], Audrey Strongosky[393], Alexander Blanski[394], Gist Croft[395], Lisa Voltolina[396], Whitley Aamodt[397], Stewart A Factor[398], Nabila Dahodwala[399], Chantale Branson[400], Krutika Patel[401], Shyamal Mehta[402], Miguel Inca Martinez[403], Anne-Marie Wills[404], Ejaz A. Shamim[405], Charles H. Adler[406], Peter Heutink[407], Duan Nguyen[408], Toan Nguyen[408], NguyễN Thái Thuỳ Ngân[409], Ha Ngoc Le Uyen[409], Tai Ngoc Tran[409], Khang Yo[409], Vinh Thanh Nguyen[409], Masharip Atadzhanov[410]

[1] Department of Neurodegenerative Diseases, Hertie Institute for Clinical Brain Research, University of Tuebingen and German Center for Neurodegenerative Diseases (DZNE), Tuebingen, Germany
[2] Department of Neurology, Oslo University Hospital, Oslo, Norway
[3] Centre Hospitalo-Universitaire Dr Benbadis Constantine, Constantine, Algeria
[4] Hospital university of Oran-Algeria, Oran, Algeria
[5] Faculty of Biological Sciences, USTHB Bab Ezzouar, Algiers, Algeria
[6] Sanatorio de la Trinidad Mitre- INEBA, Buenos Aires, Argentina
[7] Hospital JM Ramos Mejia, Buenos Aires, Argentina
[8] Centro de Educación Médica e Investigaciones Clínicas Norberto Quirno, Buenos Aires, Argentina
[9] Hospital General San Martin, La Plata, Argentina
[10] Hospital Fernandez, Buenos Aires, Argentina
[11] HOSPITAL NACIONAL PROFESOR ALEJANDRO POSADAS, Buenos Aires, Argentina

[12] CONICET-UNT, San Miguel de Tucumán, Argentina
[13] Somnus Neurology Clinic, Yerevan, Armenia
[14] Neuroscience Research Australia, Sydney, Australia
[15] Garvan Institute of Medical Research and Concord Repatriation General Hospital, Darlinghurst, Australia
[16] Concord Hospital, Concord, Australia
[17] QIMR Berghofer Medical Research Institute, Herston, Australia
[18] Murdoch University, Perth, Australia
[19] QIMR Berghofer Medical Research Institute, Brisbane, Australia
[20] Epworth hospital, Melbourne, Australia
[21] Garvan Institute of Medical Research, Sydney, Australia
[22] Royal North Shore Hospital, Sydney, Australia
[23] University of Sydney, Sydney, Australia
[24] Flinders Medical Centre, Bedford Park, Australia
[25] Perron Institute for Neurological and Translational Science, Nedlands, Australia
[26] Perron Institute, Perth, Australia
[27] Sydney Local Health District, Sydney, Australia
[28] Westmead Hospital, Westmead, Australia
[29] MonoPD, Sydney, Australia
[30] Florey neuroscience, Melbourne, Australia
[31] Westmead Hospital, Sydney, Australia
[32] Central Adelaide Local Health Network, Adelaide, Australia
[33] St Vincent's Hospital Sydney, Darlinghurst, Australia
[34] University of Queensland, Brisbane, Australia
[35] Perron Institute of Neurological and Translational Science, Perth, Australia
[36] Concord Repatriation General Hospital, Sydney, Australia
[37] Medical University Vienna Austria, Vienna, Austria
[38] Istanbul Klinik, Baku, Azerbaijan
[39] Istanbul clinic Movement disorders center, Baku, Azerbaijan
[40] National Institute of Neurosciences and Hospital, Dhaka, Bangladesh
[41] University of Antwerp, Antwerp, Belgium
[42] Universidade Federal do Rio Grande do Sul / Hospital de Clínicas de Porto Alegre, Porto Alegre, Brazil
[43] Federal University of Health Sciences of Porto Alegre, Porto Alegre, Brazil
[44] Universidade Federal do Rio Grande do Sul, Porto Alegre, Brazil
[45] University of São Paulo, São Paulo, Brazil
[46] Universidade Federal de Minas Gerais, Belo Horizonte, Brazil
[47] Federal University of Ceará, Fortaleza, Brazil
[48] Institut universitaire de gériatrie de Montréal, Montreal, Canada
[49] McGill University, Montreal, Canada
[50] Aligning Science Across Parkinson's, Vancouver, Canada
[51] University of Toronto, Toronto, Canada
[52] Fundación Diagnosis, Santiago, Chile
[53] Faculty of Medicine Universidad de Chile, Santiago, Chile

[54] Universidad de Chile - Clínica Alemana Santiago, Santiago, Chile
[55] Universidad Católica del Maule, Talca, Chile
[56] University of Chile, Santiago, Chile
[57] Universidad de Chile, Santiago, Chile
[58] Universidad del Desarrollo, Santiago, Chile
[59] Universidad de Santiago de Chile, Santiago, Chile
[60] Hospital San Juan de Dios, La serena, Chile
[61] Inmov, Santiago, Chile
[62] Universidad de Chile, Facultad de Medicina, Santiago, Chile
[63] University of Concepción, Concepcion, Chile
[64] Centro de Imagenología, Hospital Clínico Universidad de Chile, Santiago, Chile
[65] Central South University, Changsha, China
[66] West China Hospital Sichuan University, Chengdu, China
[67] Xiangya Hospital, Changsha, China
[68] Capital Medical University, Beijing, China
[69] Zhejiang University, Hangzhou, China
[70] Xiangya Hospital, Central South University, Changsha, China
[71] Queen Elizabeth Hospital, Kowloon, China
[72] The Hong Kong University of Science and Technology, Kowloon, China
[73] Universidad Nacional de Colombia, Bogotá, Colombia
[74] Fundación Valle del Lili, Santiago De Cali, Colombia
[75] GRUPO DE NEUROCIENCIAS DE ANTIOQUIA (GNA), Medellín, Colombia
[76] Universidad de Antioquia, Medellín, Colombia
[77] University of Costa Rica, San Jose, Costa Rica
[78] Aarhus University, Aarhus, Denmark
[79] The American University in Cairo, Cairo, Egypt
[80] Beni-Suef University, Beni Suef, Egypt
[81] Dr. Andres Bello university, San Salvador, El Salvador
[82] Universidad Dr Andrés Bello, San Salvador, El Salvador
[83] UNAB, San Salvador, El Salvador
[84] Addis Ababa University, Addis Ababa, Ethiopia
[85] Paris Brain Institute, Paris, France
[86] Sorbonne Université, Paris, France
[87] Salpêtrière Hospital (AP-HP), Sorbonne Université, Paris, France
[88] University Hospital of Strasbourg, Strasbourg, France
[89] Paris Brain Institute - Sorbonne University, Paris, France
[90] université Toulouse, toulouse, France
[91] chu toulouse, toulouse, France
[92] Paris Brain Institute (ICM), Paris, France
[93] CHU Lille, Lille, France
[94] Institut du Cerveau-Paris Brain Institute-ICM, Paris, France
[95] Nantes Université, Nantes, France
[96] University of Bordeaux, Bordeaux, France
[97] Hospices civils de Lyon, Hopital Neurologique Pierre Wertheimer, BRON, France

[98] Limoges University Hospital, Limoges, France
[99] CHU Toulouse, TOULOUSE, France
[100] CIC Neurosciences, Paris Brain institute, Paris, France
[101] Tbilisi State Medical University, Tbilisi, Georgia
[102] S. Khechinashvili University Hospital, Tbilisi, Georgia
[103] Ivane Javakhishvili Tbilisi State University, Tbilisi, Georgia
[104] Ilia State University, Tbilisi, Georgia
[105] University of Lübeck, Lübeck, Germany
[106] Deutsches Zentrum für Neurodegenerative Erkrankungen, Göttingen, Germany
[107] University Medical Center Göttingen, Göttingen, Germany
[108] Department of Neurology, University Hospital, LMU Munich, Munich, Germany
[109] University of Tubingen, Tübingen, Germany
[110] University of Mainz, Mainz, Germany
[111] The German Center for Neurodegenerative Diseases, Göttingen, Germany
[112] Charité - Universitätsmedizin Berlin, Berlin, Germany
[113] University Medical Center Schleswig-Holstein, Lübeck, Germany
[114] Technical University of Munich, Munich, Germany
[115] German Centre for Neurodegenerative Diseases (DZNE) / University Hospital Bonn, Bonn, Germany
[116] University Hospital Bonn, Bonn, Germany
[117] Hannover Medical School, Hannover, Germany
[118] UKS, University of Saarland, Homburg and Mainz, Germany
[119] CENTOGENE, Rostock, Germany
[120] Centogene GmbH, Berlin, Germany
[121] Kiel University, Kiel, Germany
[122] Centogene GmbH, Rostock, Germany
[123] University of Ghana Medical School, Accra, Ghana
[124] Richard Novati Catholic Hospital, Catholic Health Service Trust, Accra, Ghana
[125] Kwame Nkrumah University of Science and Technology, Kumasi, Ghana
[126] University of Thessaly, Volos, Greece
[127] Aristotle University of Thessaloniki, Thessaloniki, Greece
[128] Ionian University, Corfu, Greece
[129] Biomedical research Foundation of the Academy of Athens, Athens, Greece
[130] Diagnostic and Therapeutic Centre HYGEIA Hospital, Marousi, Greece
[131] University of Ioannina, Ioannina, Greece
[132] University of Crete, Heraklion, Greece
[133] HYGEIA Hospital, Athens, Greece
[134] Hospital San Felipe, Tegucigalpa, Honduras
[135] Universidad Tecnológica Centroamericana (UNITEC), Tegucigalpa, Honduras
[136] Universidad Tecnológica Centroamericana UNITEC, Tegucigalpa, Honduras
[137] Universidad Tecnológica Centroamericana, Tegucigalpa, Honduras
[138] Fundación Lucas para la Salud, Tegucigalpa, Honduras
[139] deCODE genetics/Amgen Inc., Reykjavik, Iceland Faculty of Medicine, University of Iceland, Reykjavik, Iceland

[140] Aster Medcity, Kochi, India
[141] Sree Chitra Tirunal Institute for Medical Sciences and Technology, Thiruvananthapuram, India
[142] National Institute of Mental Health & Neurosciences, Bengaluru, India
[143] Manipal Hospital, Delhi, India
[144] All India Institute of Medical Sciences, Delhi, India
[145] Nizam's Institute Of Medical Sciences, Hyderabad, India
[146] Shahid Beheshti University of Medical Science, Tehran, Iran
[147] Tel Aviv Sourasky Medical Center, Tel Aviv-Yafo, Israel
[148] Tel Aviv Medical Center, Tel Aviv-Yafo, Israel
[149] Magna Graecia University of Catanzaro, Catanzaro, Italy
[150] University of Pavia, Pavia, Italy
[151] National Research Council, Cosenza, Italy
[152] University of Perugia, Perugia, Italy
[153] Magna Graecia University, Catanzaro, Italy
[154] University of Rome Tor Vergata, Rome, Italy
[155] IRCCS Mondino Foundation, Pavia, Italy
[156] University of Naples Federico II, Naples, Italy
[157] IRCCS Neuromed, Pozzilli, Italy
[158] Juntendo University, Tokyo, Japan
[159] Juntendo University faculty of medicine, Tokyo, Japan
[160] Jikei University School of Medicine, Tokyo, Japan
[161] Juntendo University, Bunkyō, Japan
[162] Institute of Neurology and Neurorehabilitation, Almaty, Kazakhstan
[163] West Kazakhstan Marat Ospanov State Medical University, Aktobe, Kazakhstan
[164] Medline medical center, Astana, Kazakhstan
[165] National Center for Neurosurgery, Astana, Kazakhstan
[166] Astana Medical University, Astana, Kazakhstan
[167] International University of Postgraduate Education, Almaty, Kazakhstan
[168] South Kazakhstan Medical Academy, Shymkent, Kazakhstan
[169] International Research Institute of Postgraduate Education, Almaty, Kazakhstan
[170] Semey Medical University, Semey, Kazakhstan
[171] Kyrgyz State Medical Academy, Bishkek, Kyrgyzstan
[172] University of Luxembourg, Esch-sur-Alzette, Luxembourg
[173] University of Malaya, Kuala Lumpur, Malaysia
[174] Universiti Kebangsaan Malaysia, Selangor, Malaysia
[175] UKM Medical Molecular Biology Institute, Kuala Lumpur, Malaysia
[176] Universiti Kebangsaan Malaysia Medical Centre, Kuala Lumpur, Malaysia
[177] International Islamic University, Kuala Lumpur, Malaysia
[178] Hospital Queen Elizabeth, Kota Kinabalu, Malaysia
[179] HOSPITAL SULTANAH NUR ZAHIRAH KUALA TERENGGANU, KUALA TERENGGANU, Malaysia
[180] Hospita Kuala Lumpur, Kuala Lumpur, Malaysia
[181] Island Hospital, Penang, Malaysia

[182] Tecnologico de Monterrey, Monterrey, Mexico
[183] Universidad Nacional Autónoma de México, Santiago de Querétaro, Mexico
[184] Instituto Nacional de Neurología y Neurocirugía, Mexico City, Mexico
[185] Centro Neurológico del Centro Médico ABC, Campus Santa Fe, Mexico City, Mexico
[186] UNIVERSITY HOSPITAL "DR JOSE E GONZALEZ", Monterrey, Mexico
[187] Hospital Ángeles Puebla, Puebla, Mexico
[188] Hospital Ángeles Puebla, Universidad Anáhuac Puebla, Puebla, Mexico
[189] ISSSTE Morelia, Morelia, Mexico
[190] HOSPITAL GENERAL DE MEXICO, Mexico City, Mexico
[191] Hospital Angeles de Puebla, Puebla, Mexico
[192] Instituto Nacional de Neurología y Neurocirugía Manuel Velasco Suárez, Mexico City, Mexico
[193] Universidad Nacional Autonoma de Mexico, Mexico City, Mexico
[194] National Autonomous University of Mexico, Mexico City, Mexico
[195] Mongolian National University of Medical Sciences, Ulaanbaatar, Mongolia
[196] Specialities Hospital, CHU Ibn Sina, Rabat, Morocco
[197] Clinique OCEANIC, CASABLANCA, Morocco
[198] Tribhuvan University, Kirtipur, Nepal
[199] Vanderbilt University Medical Center, Amsterdam, Netherlands
[200] Radboud University, Nijmegen, Netherlands
[201] Radboud University Medical Center, Nijmegen, Netherlands
[202] Brain Research and Innovation Center, Amsterdam, Netherlands
[203] University of Otago, Dunedin, New Zealand
[204] New Zealand Brain Research Institution, Christchurch, New Zealand
[205] University of Canterbury, Christchurch, New Zealand
[206] University of Lagos, Lagos, Nigeria
[207] College of Medicine of the University of Lagos, Lagos, Nigeria
[208] University of Calabar Teaching Hospital, Calabar, Nigeria
[209] University of Ilorin, Ilorin, Nigeria
[210] General Hospital, Lagos, Nigeria
[211] Ahmadu Bello University, Kaduna State, Nigeria
[212] University of Abuja Teaching Hospital, Gwagwalada, Nigeria
[213] Jos University Teaching Hospital, Jos, Nigeria
[214] College of Medicine, University of Lagos, Lagos, Nigeria
[215] Lagos University Teaching Hospital, Lagos, Nigeria
[216] Neuroscience and Ageing Research Unit, Institute for Advanced Medical Research and Training, College of N, Ibadan, Nigeria
[217] Asokoro District Hospital, Abuja, Nigeria
[218] Delta State University, Abraka, Nigeria
[219] University of Ilorin Teaching Hospital, Ilorin, Nigeria
[220] Irrua Specialist Teaching Hospital, Ilorin, Nigeria
[221] University College Hospital, Ibadan, Nigeria
[222] Federal University of Health Sciences Teaching Hospital, Azare, Nigeria
[223] Obafemi Awolowo University Teaching Hospitals Complex, Ile-Ife, Nigeria
[224] University of Benin, Benin City, Nigeria

[225] Benue State University, Makurdi, Nigeria
[226] Obafemi Awolowo University, Ile-Ife, Nigeria
[227] University of Port Harcourt Teaching Hospital, Port Harcourt, Nigeria
[228] Nnamdi Azikiwe University Teaching Hospital, Nnewi, Nigeria
[229] Rivers State University Teaching Hospital, Port Harcourt, Nigeria
[230] University of Maiduguri Teaching Hospital, Maiduguri, Nigeria
[231] Federal University of Health Sciences, Otukpo, Nigeria
[232] Federal Medical Center, Owo, Nigeria
[233] Lagos State University Teaching Hospital, Ikeja, Nigeria
[234] College of Medicine, University of Lagos & R-Jolad Hospital, Lagos, Nigeria
[235] University of Nigeria Teaching Hospital, Ituku-Ozalla, Nigeria
[236] University of Abuja, Abuja, Nigeria
[237] University of Port Harcourt, Port Harcourt, Nigeria
[238] Lagos State University College of Medicine, Ikeja, Nigeria
[239] Federal Medical Center, Lagos, Nigeria
[240] Bayero University Kano, Kano, Nigeria
[241] National Hospital, Abuja, Nigeria
[242] Federal Teaching Hospital, Gombe, Nigeria
[243] Stavanger University Hospital, Stavanger, Norway
[244] University of Science and Technology Bannu, Bannu, Pakistan
[245] Razi Hospital, Rawalpindi, Pakistan
[246] Universidad Cientifica del Sur, Lima, Peru
[247] Metropolitan Medical Center, Manila, Philippines
[248] Chong Hua Hospital, Cebu, Philippines
[249] Medical University of Lodz, Lodz, Poland
[250] Research Center of Neurology, Moscow, Russia
[251] Ufa Federal Research Center, Ufa, Russia
[252] Ufa Scientific Center, Ufa, Russia
[253] Russian Academy of Sciences / Bashkir State Medical University, Ufa, Russia
[254] King Faisal Specialist Hospital and Research Center, Riyadh, Saudi Arabia
[255] King Abdullah International Medical Research Center, Jeddah, Saudi Arabia
[256] Neurology Clinic, University Clinical Center of Serbia, Belgrade, Serbia
[257] National Neuroscience Institute, Singapore, Singapore
[258] Nanyang Technological University, Singapore, Singapore
[259] Ljubljana University Medical Centre, Ljubljana, Slovenia
[260] University of KwaZulu-Natal, Durban, South Africa
[261] University of Stellenbosch, Stellenbosch, South Africa
[262] Stellenbosch University, Stellenbosch, South Africa
[263] University of the Western Cape, Bellville, South Africa
[264] Stellenbosch University, Cape Town, South Africa
[265] University of Pretoria, Pretoria, South Africa
[266] Seoul National University Hospital, Seoul, South Korea
[267] Yongin Severance Hospital, Seoul, South Korea
[268] Seoul National University, Seoul, South Korea

[269] SMG-SNU Boramae Medical Center, College of Medicine Seoul National University, Seoul, South Korea
[270] Hospital Universitario Burgos, Burgos, Spain
[271] University Hospital Mutua Terrassa, Barcelona, Spain
[272] Institut de Recerca Sant Joan de Deu, Barcelona, Spain
[273] Research Institute Germans Trias i Pujol, Barcelona, Spain
[274] Instituto de Biomedicina de Sevilla, Seville, Spain
[275] University Hospital Germans Trias i Pujol, Barcelona, Spain
[276] Hospital Clínic de Barcelona, Barcelona, Spain
[277] FCRB-IDIBAPS, Barcelona, Spain
[278] Fernando Pessoa University, San Roque Hospital, Las Palmas de Gran Canaria, Spain
[279] Hospital Universitario San Roque Las Palmas/ Universidad Fernando Pessoa Canarias (UFPC), Las Palmas de Gran Canaria, Spain
[280] Hospital Ramón y Cajal, Madrid, Spain
[281] Hospital Universitario Infanta Sofia, Madrid, Spain
[282] Hospital Universitari Vall d'Hebron, Barcelona, Spain
[283] Hospital Universitario Fundación Alcorcón, Madrid, Spain
[284] Hospital Universitario Central de Asturias, Oviedo, Spain
[285] IDIBAPS / Hospital Clinic, Barcelona, Spain
[286] IDIBAPS-FCRB. Hospital Clinic Barcelona, Barcelona, Spain
[287] Hospital Clinic de Barcelona. Institut d'Investigacio Biomedica August Pi i Sunyer (IDIBAPS), Barcelona, Spain
[288] Universidad Fernando Pessoa Canarias, Las Palmas de Gran Canaria, Spain
[289] IR Sant Pau, Barcelona, Spain
[290] Faculty of Medicine, University of Khartoum, Khartoum, Sudan
[291] Lund University, Lund, Sweden
[292] Karolinska Institute, Stockholm, Sweden
[293] Inselspital Bern, University of Bern, Bern, Switzerland
[294] University Hospital Bern, Bern, Switzerland
[295] National Taiwan University Hospital, Taipei City, Taiwan
[296] Chang Gung Memorial Hospital, Taoyuan City, Taiwan
[297] National Taiwan University, Taipei City, Taiwan
[298] National Taiwan University Hospital, Taipei, Taiwan
[299] Avicenna Tajik State Medical University, Dushanbe, Tajikistan
[300] Mongi Ben Hmida National Institute of Neurology, Tunis, Tunisia
[301] Habib Bourguiba University Hospital, Sfax, Tunisia
[302] Razi Hospital, Tunis, Tunisia
[303] Koç University, Istanbul, Turkey
[304] University of Ankara, Ankara, Turkey
[305] Şişli Etfal Training and Research Hospital, University of Health Sciences, Istanbul, Turkey
[306] Private Practice, Ankara, Turkey
[307] Istanbul University, Faculty of Medicine, Istanbul, Turkey
[308] Istanbul University, Istanbul, Turkey

[309] Istanbul Faculty of Medicine, Istanbul, Turkey
[310] Istanbul University-Cerrahpasa, Cerrahpasa Faculty of Medicine, Istanbul, Turkey
[311] Queen Mary University of London, London, United Kingdom
[312] University College London, London, United Kingdom
[313] University of Plymouth, Plymouth, United Kingdom
[314] University of Glasgow, Glasgow, United Kingdom
[315] Cardiff University, Cardiff, United Kingdom
[316] University of Bristol, Bristol, United Kingdom
[317] St George's, University of London, London, United Kingdom
[318] University of Cambridge, Cambridge, United Kingdom
[319] University of Oxford, Oxford, United Kingdom
[320] Northumbria Healthcare at NHS Foundation Trust, Newcastle upon Tyne, United Kingdom
[321] Imperial College London, London, United Kingdom
[322] Newcastle University, Newcastle upon Tyne, United Kingdom
[323] YLD, London, United Kingdom
[324] University of Manchester, Manchester, United Kingdom
[325] UCL Queen Square Institute of Neurology, London, United Kingdom
[326] University College London, Institute of Neurology, London, United Kingdom
[327] Queen Mary University of London / Guy's and St Thomas' NHS Foundation Trust, London, United Kingdom
[328] Laboratory of Neurogenetics, National Institute on Aging/Institute of Neurogenetics, University of Luebeck, Bethesda, USA
[329] Broad Institute of MIT and Harvard, Cambridge, USA
[330] University of Puerto Rico, San Juan, USA
[331] The Michael J. Fox Foundation for Parkinson's Research, New York, USA
[332] Global Parkinson's Genetics Program (GP2), Bethesda, USA
[333] Augusta University / University of Georgia Medical Partnership, Augusta, USA
[334] Mid-Atlantic Permanente Medical Group, Bethesda, USA
[335] Washington University, St. Louis, USA
[336] Aligning Science Across Parkinson's (ASAP), Bethesda, USA
[337] Indiana University, Bloomington, USA
[338] Data Tecnica, Bethesda, USA
[339] Rush University, Chicago, USA
[340] National Institutes of Health, Bethesda, USA
[341] Aligning Science Across Parkinson's (ASAP), Washington, USA
[342] Banner Sun Health Research Institute, Sun City, USA
[343] Data Tecnica, Washington, USA
[344] Michigan State University, East Lansing, USA
[345] Cleveland Clinic, Cleveland, USA
[346] Northwestern University, Evanston, USA
[347] Kaiser Permanente, Oakland, USA
[348] Baylor College of Medicine, Bethesda, USA
[349] Baylor College of Medicine, Houston, USA
[350] Baylor College of Medicine / Texas Children's Hospital, Houston, USA

[351] Parkinson's Foundation, Princeton, USA
[352] University of Miami Miller School of Medicine, Miami, USA
[353] Beth Israel Deaconess Medical Center, Boston, USA
[354] North Shore University Health System, Chicago, USA
[355] Institute for Neurodegenerative Disorders, New Haven, USA
[356] University of Pittsburgh, Pittsburgh, USA
[357] University of Alabama at Birmingham, Birmingham, USA
[358] University of Maryland, Baltimore, USA
[359] University of Cincinnati, Cincinnati, USA
[360] Northwestern University, Chicago, USA
[361] Universit of Michigan, Ann Arbor, USA
[362] Columbia University, New York, USA
[363] James J. Peters Veterans Affairs Medical Center, New York, USA
[364] University of Chicago, Chicago, USA
[365] Indiana University School of Medicine, Indianapolis, USA
[366] Aligning Science Across Parkinson's (ASAP), Baltimore, USA
[367] Gladstone Institutes, San Francisco, USA
[368] Icahn School of Medicine at Mount Sinai, New York, USA
[369] Banner Health, Phoenix, USA
[370] Aligning Science Across Parkinson's (ASAP), Vancouver, USA
[371] The Queen's Medical Center, Honolulu, USA
[372] University of Florida College of Medicine, Gainesville, USA
[373] The University of Alabama at Birmingham Heersink School of Medicine, Birmingham, USA
[374] LSU Health Shreveport, Shreveport, USA
[375] University of California, Berkeley, Berkeley, USA
[376] NYU Grossman School of Medicine, New York, USA
[377] Mayo Clinic College of Medicine, Rochester, USA
[378] VA Puget Sound Health Care System, Seattle, USA
[379] National Institutes of Health, Rockville, USA
[380] University of California, Los Angeles, Los Angeles, USA
[381] Mayo Clinic, Rochester, USA
[382] Barrow Neurological Institute, Phoenix, USA
[383] Mayo Clinic AZ, Scottsdale, USA
[384] Endeavor Health (formerly NorthShore University Health System), Skokie, USA
[385] Mayo Clinic, Jacksonville, USA
[386] The Ohio State University Medical Center, Columbus, USA
[387] The Ohio State University, Columbus, USA
[388] Parkinson's Foundation, New York, USA
[389] UAB, Birmingham, USA
[390] U. Pennsylvania, Philadelphia, USA
[391] University of Michigan, Ann Arbor, USA
[392] Van Andel Institute, Grand Rapids, USA
[393] Mayo Clinic Florida, Jacksonville, USA
[394] Van Andel Research Institute, Grand Rapids, USA

[395] The New York Stem Cell Foundation, New York, USA
[396] New York Stem Cell Foundation Reseach Institute, New York, USA
[397] University of Pennsylvania, Philadelphia, USA
[398] Emory University, Atlanta, USA
[399] University of Pennsyvlania, Philadelphia, USA
[400] Morehouse School of Medicine, Atlanta, USA
[401] New York Stem Cell Foundation, Denver, USA
[402] Mayo Clinic, Arizona, Scottsdale, USA
[403] Cleveland Clinic Foundation, Cleveland, USA
[404] Massachusetts General Hospital, Boston, USA
[405] Kaiser Permanente, MidAtlantic Permanente Research Institute, Washington, USA
[406] Mayo Clinic College of Medicine, Mayo Clinic Arizona, Scottsdale, USA
[407] Global Parkinson's Genetics Program (GP2), Pacifica, USA
[408] Hue University, Hué, Vietnam
[409] University Medical Center, Ho Chi Minh City, Vietnam
[410] University of Zambia, Lusaka, Zambia

NS-PARK Consortium (Part of GP2)

Silvia DI LEGGE[1], Lucy FAMER[1], Melissa TIR[2], Mickael AUBIGNAT[2], Astrid CAUSEL[2], Lydie ROMEO[2], Constance BISSESSUR[2], Bertrand DEGOS[3], Marie MONGIN[3], Arnaud LAPOSTOLLE[3], Lucie BRACCAGNI[3], Hiba SIFAOUI[3], Juliette PALISSON[3], Kenza BENRAHMOUNE IDRISSI[3], Thierry MOULIN[4], Matthieu BEREAU[4], Gautier CLEMENT[4], Audace CURE-MARTIN[4], Wassilios MEISSNER[5], Alexandra FOUBERT-SAMIER[5], Brice LAURENS[5], Sylvain VERGNET[5], Thomas BORAUD[5], David BENDETOWICZ[5], Jade SARRABERE[5], Dominique GUEHL[5], Pierre BURBAUD[5], Edouard COURTIN[5], Sandrine DUPOUY[5], Sandrine VILLARS[5], Wei-Ho LAI[5], Claire THIRIEZ[6], Paul-Alexandre PFEIFFER[6], Gilles DEFER[6], Rachida BARI[6], Damien CHEVANNE[6], Ana MARQUES[7], Bérengère DEBILLY[7], Philippe DEROST[7], Charlotte BEAL[7], Elodie DURAND[7], Isabelle RIEU[7], Stephane BERNARD[7], Corinne GARSAULT[7], Nathalie MEUNIER[7], Philippe REMY[8], Hayet SALHI[8], Alice DORMEUIL[8], Aimée PETIT[8], Alban GRAVIER[8], Alexia CRESSON[8], Marine SGARD[8], Marie DREANO[8], Justine MONTILLOT[8], Renaud MASSART[8], Gwendoline DUPONT[9], Vincent SCHNEIDER[9], Lucie GARNIER[9], Pascale GREBENT[9], Elena MORO[10], Pierre PELISSIER[10], Luc DEFEBVRE[11], Nicolas CARRIERE[11], Valérie SANTRAINE[11], Jean-Luc HOUETO[12], Olivier COLIN[12], Philippe COURATIER[12], Thomas GAUDIN[12], Pierre BOUTET[12], Cécile THUILIER[12], Coralie CHALOT[12], Céline PREVOST[12], Hélène VIDEAUD[12], Justine PICUT[12], Christian TARRADE[12], Stéphane THOBOIS[13], Chloé LAURENCIN[13], Stephane PRANGE[13], Paul JAULENT[13], Bruno PLUS[13], Helene GERVAIS-BERNARD[13], Catherine CAIRE[13], Hélène MERLE[13], Elise METEREAU[13], Mathilde MILLOT[13], Chloe BERNARDI[13], Emilie FAVRE[13], Adelaide JAULENT[13], Jean-Philippe AZULAY[14], Alexandre EUSEBIO[14], Frédérique FLUCHERE[14], Stephan GRIMALDI[14], Valentin MIRA[14], Tatiana WITJAS[14], Laura MUNDLER[14], Blandine DUFRESNE[14], Eve BENCHETRIT[14], Christian GENY[15], Mahmoud CHARIF[15], Ophelie FORSTER[15], Alix DURAND[15], Pauline PRIN[15], Valérie DRISS[15], Alexia ARIFI[15], Maura RODRIGUES[15], Solène FRISMAND[16], Lucie HOPES[16], Amory JARDEL[16], Salome PUISIEUX[16], Guillemette CLEMENT[16], Lili LE MONNIER[16], Nathalie DUMONT[16], Virginie BABLON[16], Anne-Gaëlle CORBILLE[17], Philippe DAMIER[17], Tiphaine ROUAUD[17], Pascal DERKINDEREN[17], Arthur

LIONNET[17], Adrien DE GUILHEM DE LATAILLADE[17], Régis FRENAIS[17], Caroline HERVE[17], Christelle GUIMBER[17], Caroline GIORDANA[18], Cosmin ALECU[18], Charlotte HERAUD[18], Vanessa FERRIER[18], Elodie DAVID[18], Christina FAROUL[18], Giovanni CASTELNOVO[19], Marie DE VERDAL[19], Leslie FRA[19], Elsa FOUCARAN[19], Fatima-Ezzahra ENNAJI[19], Jean-Christophe CORVOL[20], Stephanie CARVALHO[20], Yajiththa RAJASEGARAM[20], Florence TUBACH[20], Yann DE RYCKE[20], Nathalie BERTILLE[20], David TAVEL[20], Dounya METDAOUI[20], Avigaelle ABITBOL[20], Mathias ANTUNES[20], Alexis BRICE[20], Suzanne LESAGE[20], Christelle TESSSON[20], Fanny CASSE[20], Mélanie FERRIEN[20], Guillaume COGAN[20], Lisa WELMENT[20], Sylvie FORLANI[20], Sophia LOIODICE[20], Ludmila JORNEA[20], Graziella MANGONE[20], Sara SAMBIN[20], Aymeric LANORE[20], Thomas COURTIN[20], Louise-Laure MARIANI[20], Fouad KHOURY[20], Poornima MENON[20], Florence CORMIER-DEQUAIRE[20], Emmanuel FLAMAND-ROZE[20], David GRABLI[20], Elodie HAINQUE[20], Marie VIDHAILLET[20], Aurélie MENERET[20], Cécile DELORME[20], Cendrine FOUCARD[20], Florian VON RAISON[20], Alexis ELBAZ[20], Andreas HARTMANN[20], Vincent LECLERCQ[20], Theodore SOULIER[20], Daniel TORRES[20], Giulia COARELLI[20], Giorgia QUERIN[20], Fabien HAUW[20], Margaux DUNOYER[20], Jeremy BONETTO[20], Ryad LADGHEM-CHIKOUCHE[20], Mickael LE[20], Sophie LIOT[20], Sonia MESSAR[20], Hamza SALAH[20], Amelie BERNARDO[20], Naoual SERARI[20], Isabelle BENATRU[21], Solène ANSQUER[21], Emilie RABOIS[21], Carole DAVID[21], Zoe FOURNIER[21], Anne DOE DE MAINDREVILLE[22], Guillaume CAREY[22], Margaux BONNAIRE-VERDIER[22], Elise CHEVAILLIER[22], Sophie DRAPIER[23], Frederique LEH[23], Marion LECLERCQ[23], Simon LAMY[23], Françoise KESTENS[23], Rozenn GOURHAN[23], Sandra LOPEZ-ALFARO[23], Jean-François HOUVENAGHEL[23], Mélanie ALEXANDRE[23], Christine BOURDONNAIS[23], David MALTETE[24], Guillaume COSTENTIN[24], Clemence HARDY[24], Linda VERNON[24], Ahmed BOUMEDIENE[24], Christine TRANCHANT[25], Mathieu ANHEIM[25], Ouhaid LAGHA BOUKBIZA[25], Thomas WIRTH[25], Jimmy VOIRIN[25], Marie des Neiges SANTIN[25], Thomas BOGDAN[25], Hugo RUMMEL[25], Céline JULIE[25], Nadine LONGATO[25], Clélie PHILLIPPS[25], Anne Claire ANDRIES-ROS[25], Olivier RASCOL[26], Vanessa ROUSSEAU[26], Samuel TESSIER[26], Paolo BASTOS[26], Margherita FABBRI[26], Fabienne ORY-MAGNE[26], Christine BREFEL COURBON[26], Clemence LEUNG[26], Hélène CATALA[26], Gabrielle SILL[26], Raquel PINHEIRO BARBOSA[26], Stéphanie BRAS[26], Estelle HARROCH[26], Claudia GILLET[26], Yoan HERADES[26], Eva CAMGRAND[26], Iker VAQUERO ALBA[27], Céline PEGOURIE[27]

[1] Centre Hospitalier du Pays d'Aix, Aix-en-Provence, France
[2] Centre Hospitalier Universitaire Amiens site sud, Amiens, France
[3] Hopital Avicenne, AP-HP, Bobigny, France
[4] Centre Hospitalier Regional Universitaire Jean Minjoz, Besançon, France
[5] Centre Hospitalier Universitaire de Bordeaux, Bordeaux, France
[6] Centre Hospitalier Regional Universitaire Caen, Caen, France
[7] Centre Hospitalier Universitaire Gabriel Montpied, Clermont-Ferrand, France
[8] Centre Hospitalier Universitaire Henri Mondor, AP-HP, Créteil, France
[9] Centre Hospitalier Regional Universitaire Dijon Bourgogne, Dijon, France
[10] Centre Hospitalier Universitaire Grenoble, Grenoble, France
[11] Centre Hospitalier Regional Universitaire de Lille, Lille, France
[12] Centre Hospitalier Universitaire Dupuytren, Limoges, France
[13] Hopital neurologique Pierre Wertheimer, Lyon, France
[14] Hopital de la Timone, Marseille, France

[15] Centre Hospitalier Universitaire de Montpellier, Montpellier, France
[16] Centre Hospitalier Universitaire de Nancy, Nancy, France
[17] Centre Hospitalier de Nantes, Nantes, France
[18] Centre Hospitalier Universitaire de Nice, Nice, France
[19] Centre Hospitalier Universitaire de Nimes, Nîmes, France
[20] Hopital Universitaire Pitie Salpetriere, AP-HP, Paris, France
[21] Centre Hospitalier Universitaire de Poitiers, Poitiers, France
[22] Centre Hospitalier Universitaire de Reims, Reims, France
[23] Centre Hospitalier Regional Universitaire de Rennes, Rennes, France
[24] Centre Hospitalier Universitaire de Rouen, Rouen, France
[25] Hopitaux Universitaires de Strasbourg, Strasbourg, France
[26] Centre Hospitalier Universitaire de Toulouse, Toulouse, France
[27] Centre Hospitalier Universitaire de Toulouse, France, CRA

*Corresponding author: Thomas Gasser
Email: thomas.gasser@uni-tuebingen.de
